# A Novel Small Molecule Accelerates Early Persister Regrowth and Potentiates Antibiotic Killing via MdtL–DcrB

**DOI:** 10.1111/1751-7915.70368

**Published:** 2026-05-03

**Authors:** Garin Park, Hyein Kim, Sooyeon Song

**Affiliations:** ^1^ Agriculture Convergence Technology Jeonbuk National University Jeonju‐Si Jellabuk‐Do South Korea; ^2^ Department of Animal Science Jeonbuk National University Jeonju‐Si Jellabuk‐Do South Korea

**Keywords:** antibiotic potentiation, early regrowth, envelope‐associated transport, intracellular accumulation, MdtL–DcrB axis, persister cells

## Abstract

Persister cells survive antibiotic exposure through transient tolerance, often leading to infection relapse. Because antibiotic susceptibility is restored when persisters resume growth, we sought to identify a chemical modulator that advances early regrowth and to define the pathway underlying its activity. A screen of 7040 compounds led to the discovery of bymBDZ, which shortens the lag phase and promotes early regrowth in persister‐derived *Escherichia coli* . bymBDZ significantly enhanced antibiotic killing during early treatment windows when survivors are typically retained, and this activity extended to enterohemorrhagic *E. coli* O157:H7 and multiple antibiotic classes. Genetic and functional analyses showed that bymBDZ activity required the membrane transporter MdtL and the envelope factor DcrB. bymBDZ induced *dcrB* expression and remodelled envelope‐associated transport, resulting in increased intracellular exposure to small molecules during early regrowth, as indicated by elevated dye accumulation. Consistent with this remodelling, bymBDZ promoted faster growth resumption and reinforced antibiotic killing during early regrowth. Collectively, these findings identify bymBDZ as a chemical probe that modulates persister regrowth through MdtL–DcrB‐dependent envelope transport remodelling and suggest a strategy to sensitize tolerant bacteria to antibiotics.

## Graphical abstract

bymBDZ accelerates early regrowth of persister‐derived *E. coli* and potentiates antibiotic killing. Its activity requires the MdtL–DcrB axis and is associated with envelope‐linked transport changes that increase intracellular small‐molecule exposure during recovery, thereby enhancing clearance in early treatment windows.

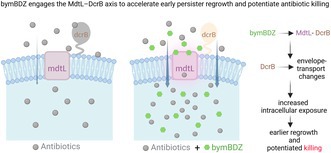

## 1 Introduction

Bacteria have evolved diverse survival strategies to endure harsh environments, including exposure to antibiotics (Harms et al. 2016; Urban‐Chmiel et al. 2022). One such strategy involves the formation of persister cells, a small subpopulation that enters a dormant, nondividing state without acquiring heritable resistance (Harms et al. 2016; Arvaniti and Skandamis 2022). Because these cells strongly downregulate metabolic activity, they can withstand otherwise lethal antibiotic treatments, resulting in a characteristic biphasic killing pattern (Fisher et al. 2017). In this pattern, the majority of actively growing cells are rapidly killed, whereas a tolerant subpopulation declines more slowly and a small fraction of persister cells survives prolonged exposure, yielding a biphasic killing curve (Song and Wood 2021). Once environmental pressures are removed, persister cells can resume metabolic activity and proliferate, contributing to chronic and recurrent infections that are notoriously difficult to eradicate (Fang and Allison 2023).

Traditional antibiotics primarily target vital processes such as protein synthesis (Forge and Schacht 2000; Arenz and Wilson 2016), cell wall biosynthesis (Schneider and Sahl 2010), and DNA replication (Pham et al. 2019), all of which require active metabolism. The dormant physiology of persisters is maintained by stress‐response programs, including toxin‐antitoxin (TA) systems and ribosome hibernation, which suppress translation and slow growth, thereby reducing antibiotic efficacy (Wang and Wood 2011; Song and Wood 2020a, 2020b). Because persisters regain susceptibility as they return to growth, accelerating the transition from dormancy to active regrowth has emerged as a promising strategy to improve antibiotic killing (Niu et al. 2024). Persister regrowth is often asynchronous and heterogeneous (Yu et al. 2019; Fang and Allison 2023); thus, shortening the lag to growth resumption can increase the fraction of cells that enter an antibiotic‐susceptible state within a defined treatment window, enhancing eradication and lowering the risk of relapse.

Growth resumption is initiated by nutrient availability and is accompanied by rapid physiological remodelling (Yamasaki et al. 2020). Persister cells sense nutrients via membrane‐associated systems such as the phosphotransferase system (PTS) and chemotaxis‐linked signalling pathways, which initiate the earliest steps of recovery (Yamasaki et al. 2020; Song et al. 2021). Dormancy is frequently associated with altered envelope properties, including reduced membrane fluidity and changes in permeability, that restrict the influx of nutrients and other small molecules (Kim et al. 2019; Menon et al. 2024). Once cells commit to growth, these physical barriers can rapidly revert, increasing uptake and restoring transport homeostasis (Stojowska‐Swędrzyńska et al. 2026). The resulting influx supports the restart of core biosynthetic processes, most notably translational reactivation through ribosome remodelling and renewed protein synthesis, which marks the transition to active regrowth (Basu and Yap 2017; Zhang et al. 2018). Nevertheless, the molecular determinants that control the kinetics of this transition remain incompletely defined. In particular, how envelope‐associated transport remodelling is coordinated with translational restart to set the timing of regrowth—and thereby restore antibiotic susceptibility within a defined treatment window—remains a key gap in current understanding.

Here, we identify bymBDZ, a hit from a 7040‐compound screen, that shortens the lag and accelerates early regrowth in persister‐derived *E. coli* , thereby potentiating antibiotic killing. Our data link this phenotype to an MdtL→DcrB axis that remodels envelope‐associated transport, increases net intracellular exposure during early regrowth, and represses envelope genes including *yraP* (*dolP*), *yegS* and *emrE*. Notably, this potentiating activity extends to enterohemorrhagic *E. coli* O157:H7 and multiple antibiotic classes, supporting broad applicability.

## 2 Materials and Methods

### 2.1 Bacteria and Growth Conditions

Bacteria (Table 1) that were used in this study were cultured routinely in lysogeny broth (Bertani 1951) at 37°C with shaking at 200 rpm. The pCA24N‐carrying strains were cultured with chloramphenicol (30 μg/mL) to retain the plasmid, and kanamycin (50 μg/mL) was used for deletion mutants.

**TABLE 1:** Strains and plasmids.

Strains and plasmids	Feature	Source
BW25113	*rrnB3 ΔlacZ47877 hsdR514 Δ(araBAD)567 Δ(rhaBAD)568 rph‐1*	Baba et al. (2006)
BW25113 Δ*dcrB*	BW25113 Δ*dcrB*, Km^R^	
BW25113 Δ*yegS*	BW25113 Δ*yegS*, Km^R^	
BW25113 Δ*yraP*	BW25113 Δ*yraP*, Km^R^	
BW25113 Δ*emrE*	BW25113 Δ*emrE*, Km^R^	
BW25113 Δ*mdtG*	BW25113 Δ*mdtG*, Km^R^	
BW25113 Δ*emrY*	BW25113 Δ*emrY*, Km^R^	
BW25113 Δ*mdtL*	BW25113 Δ*mdtL*, Km^R^	
BW25113 Δ*tsx*	BW25113 Δ*tsx*, Km^R^	
BW25113 Δ*glpT*	BW25113 Δ*glpT*, Km^R^	
ATCC 43889		Kudva et al. (1996)
MG1655‐ASV	*rrnb*P1::GFP[ASV]	Shah et al. (2006)
**Plasmids**		
pCA24N	Cm^R^; *lacI* ^*q*^	Kitagawa et al. (2005)
pCA24N_*argT*	Cm^R^; *lacI* ^*q*^, PT5‐lac:: *argT*+	
pCA24N_*cdd*	Cm^R^; *lacI* ^*q*^, PT5‐lac:: *cdd*+	
pCA24N_*cheY*	Cm^R^; *lacI* ^*q*^, PT5‐lac:: *cheY*+	
pCA24N_*cspE*	Cm^R^; *lacI* ^*q*^, PT5‐lac:: *cspE*+	
pCA24N_*dcrB*	Cm^R^; *lacI* ^*q*^, PT5‐lac:: *dcrB*+	
pCA24N_*pgsA*	Cm^R^; *lacI* ^*q*^, PT5‐lac:: *pgsA*+	
pCA24N_*trpS*	Cm^R^; *lacI* ^*q*^, PT5‐lac:: *trpS*+	
pCA24N_*ysgA*	Cm^R^; *lacI* ^*q*^, PT5‐lac:: *ysgA*+	
pCA24N_*yegS*	Cm^R^; *lacI* ^*q*^, PT5‐lac:: *yegS*+	
pCA24N_*yraP*	Cm^R^; *lacI* ^*q*^, PT5‐lac:: *yraP*+	
pCA24N_*emrE*	Cm^R^; *lacI* ^*q*^, PT5‐lac:: *emrE*+	
pCA24N_*mdtL*	Cm^R^; *lacI* ^*q*^, PT5‐lac:: *mdtL*+	

### 2.2 Persister Cells

Exponentially growing cells (turbidity 0.8 at 600 nm) were converted to persister cells by adding rifampicin (100 μg/mL) for 30 min, centrifuging, and re‐suspending into fresh LB broth containing ampicillin (100 μg/mL) for 3 h to remove non‐persister cells (Kwan Brian et al. 2013). Afterward, cells were washed with 0.85% NaCl twice.

### 2.3 Chemical Screening to Identify Persister‐Waking Chemicals

We received 7040 compounds from the Korea Chemical Bank chemical library. Chemicals were added at a concentration of 100 μM to LB broth in a 96 well plate, and persister cells were inoculated. DMSO was treated as control. Persister cell resuscitation was calculated through turbidity values at 600 nm, at 6 and 18 h.

### 2.4 Ampicillin Time‐Kill Curves

Exponential‐phased cultures were obtained by diluting overnight cultures (16 h) 1:100 in fresh LB media and grown to the turbidity 0.8 at 600 nm. Cultures were centrifuged at 4000 × *g* for 10 min, and LB or M9/0.4% glucose broth containing ampicillin (100 μg/mL) were re‐suspended. Compounds or DMSO were supplied at the concentration of 100 μM. At incubation time 0, 3, 6, 18 h, viable cells were counted by dropping 10 μL triplicate on LB agar plate.

### 2.5 Pooled ASKA Screening

To identify proteins responsible for resuscitation, all 4267 ASKA clones (GFP‐) (Kitagawa et al. 2005) were combined, grown to a turbidity of 2 at 600 nm in LB medium, and their plasmids isolated. Pooled ASKA plasmids (1uL containing 50 ng DNA) were electroporated into 50uL of *E. coli* BW25113 component cells, 1 mL of LB was added immediately, and the cells were grown to a turbidity of 0.5 at 600 nm. Chloramphenicol (30 μg/mL) was added to maintain plasmid and incubated with shaking at 200 rpm at 37°C until the turbidity at 600 nm reached 0.8. Rifampicin (100 μg/mL) was treated for 30 min, and ampicillin was applied for 3 h to remove non‐persister cells. After washing the cells with 0.85% NaCl twice, 100 μL of persister cells were plated onto M9/0.4% glucose agar containing bymBDZ. Colonies that appeared quickly were selected and sequenced (pCA24N‐F: GCCCTTTCGTCTTCACCTCG).

### 2.6 Active 70s Ribosome Assay

Persister cell of *E. coli* MG1655 *rrnbP1*::GFP[ASV] was formed as previously described, resuscitated on M9/glucose (0.4%) agarose gel pad containing DMSO or bymBDZ (100 μM), and their GFP signal was monitored using a fluorescence microscope (Zeiss Axioscope.A5). *E. coli* K‐12 MG1655‐ASVGFP expresses an unstable variant of GFP (half‐life < 1 h) under the control of the 16S rRNA ribosomal promoter *rrnBP1* (Shah et al. 2006).

### 2.7 qRT‐PCR

Quantitative real‐time PCR was conducted to (i) identify genes upregulated by bymBDZ, and (ii) investigate the transcriptional impact of *dcrB* overexpression on its regulatory network. For the first experiment, *E. coli* BW25113 wild‐type cells in exponential phase were converted to persister cells and subsequently resuscitated in M9 minimal medium supplemented with 0.4% glucose and 100 μM bymBDZ for 30 min.

For the second experiment, the related genes were selected based on STRING database (Szklarczyk et al. 2023), genomic proximity and previously reported findings (Pushpker et al. 2022). *E. coli* BW25113 strains harbouring either empty pCA24N or pCA24N‐*dcrB* were cultured in LB broth supplemented with chloramphenicol (25 μg/mL) until reaching an OD_600_ of 0.2. Expression of *dcrB* was induced by the addition of 1 mM isopropyl‐β‐D‐thiogalactopyranoside (IPTG), followed by a 30‐min induction and an additional 60‐min incubation. Total RNA was extracted using the Inclone RNA Mini Extraction Kit according to the manufacturer's instructions. qRT‐PCR were performed using the 1step AccuPower GreenStar RT‐qPCR Master Mix (Bioneer) on the CFX‐96 Real‐Time PCR Detection System (Bio‐Rad). Primer annealing was conducted at 55°C. Gene‐specific primers used in this study are listed in (Table S1). Relative gene expression was calculated using the 2^−ΔΔ*CT*^ method with *purM* as the internal reference gene. Primer specificity was confirmed by endpoint PCR.

### 2.8 Accumulation Assays of H33342

The Hoechst 33342 bis‐benzamide assay was performed as previously described (Coldham et al. 2010) with minor modifications. *E. coli* cells were cultured to an optical density at 600 nm (OD_600_) of 0.8, harvested by centrifugation at 4000 × *g* for 10 min, washed twice with 0.85% NaCl, and resuspended in phosphate‐buffered saline (PBS). The cell suspension was adjusted to an OD_600_ of 0.3 in PBS, and 176 μL aliquots were transferred to 96‐well plates. Cells were treated with DMSO, bymBDZ or carbonyl cyanide m‐chlorophenyl hydrazone (CCCP) to a final concentration of 100 μM to assess and compare efflux‐inhibiting effects. H33342 was added to each well to achieve a final concentration of 2.5 μM. Fluorescence measurements were recorded from the top of the wells using excitation and emission wavelengths of 360 and 460 nm, respectively, for 9 cycles with a 15‐min interval between cycles and a gain multiplier of 60. Two independent cultures were used, and each sample was analysed in duplicate.

### 2.9 Protein Docking Analysis

bymBDZ structures were downloaded from PubChem in 3D SDF format, converted to Mol2 using UCSF Chimera, and then saved as PDBQT files via AutoDockTools (Trott and Olson 2010). Candidate *E. coli* membrane proteins were obtained from the Protein Data Bank (Berman et al. 2000) or Alphafold (Jumper et al. 2021) in PDB format, and converted to PDBQT using AutoDockTools. Protein centres were defined in AutoDockTools, and binding pockets were predicted using POCASA (Yu et al. 2010). Docking was performed using AutoDock Vina (vina.exe) in the command prompt with two grid sizes (60 × 60 × 60 Å, spacing 0.6 Å; 24 × 24 × 24 Å, spacing 0.375 Å) (Trott and Olson 2010). Predicted binding affinities and docking poses were obtained from the output PDBQT files. Candidates not located near predicted pockets were excluded. Proteins not located near predicted binding pockets were excluded. Selection was based on two criteria: (i) a lowest binding affinity of ≤ −7 kcal/mol among the eight binding poses, and (ii) a minimal difference between the highest and lowest binding affinities. Binding conformations and protein–ligand interactions were visualized using Discovery Studio (BIOVIA).

### 2.10 Time‐Kill Curves of bymBDZ in Combination With Other Antibiotics

Exponential‐phase cultures of *E. coli* BW25113 were harvested, centrifuged at 4000 × *g* for 10 min, and resuspended in LB or M9/glucose (0.4%) with antibiotics. Concentration of all antibiotics was set above 10× the minimum inhibitory concentration (MIC) to prevent the acquisition of resistance after 18 h of incubation (ciprofloxacin, 5 μg/mL; amoxicillin, 100 μg/mL; tetracycline, 100 μg/mL; kanamycin, 200 μg/mL) (Kwan et al. 2015).

### 2.11 MIC Assay

To determine the MIC_50_ and MIC_100_ of ampicillin for *E. coli* BW25113 wild‐type and Δ*dcrB*, and to evaluate the potentiating effect of bymBDZ on ampicillin susceptibility, 5 × 10^5^ CFU/mL of exponential cells were inoculated and grown for 24 h in LB media supplemented with either DMSO or bymBDZ (100 μM) across a range of ampicillin concentrations. The MIC_100_ was defined as the lowest concentration of ampicillin that allows 100% growth. The MIC_50_ was defined as the concentration corresponding to a 50% reduction in OD_600_ relative to the control (0 μg/mL ampicillin), which was determined for each replicate by linear interpolation (Soothill et al. 1992).

### 2.12 Statistical Analysis

Statistical analyses were performed using Student's *t*‐test or two‐way ANOVA with GraphPad Prism 10 (San Diego, CA, USA). Statistical significance was defined as *p* < 0.05 (*), *p* < 0.01 (**) and *p* < 0.001 (***).

## 3 Results

### 3.1 bymBDZ (5‐[2‐(4‐Butan‐2‐Ylphenoxy)Ethyl]‐3‐Methyl‐2,3‐Dihydro‐1H‐1,5‐Benzodiazepin‐4‐One) Accelerates Persister Regrowth and Potentiates Ampicillin Killing

**FIGURE 1:**
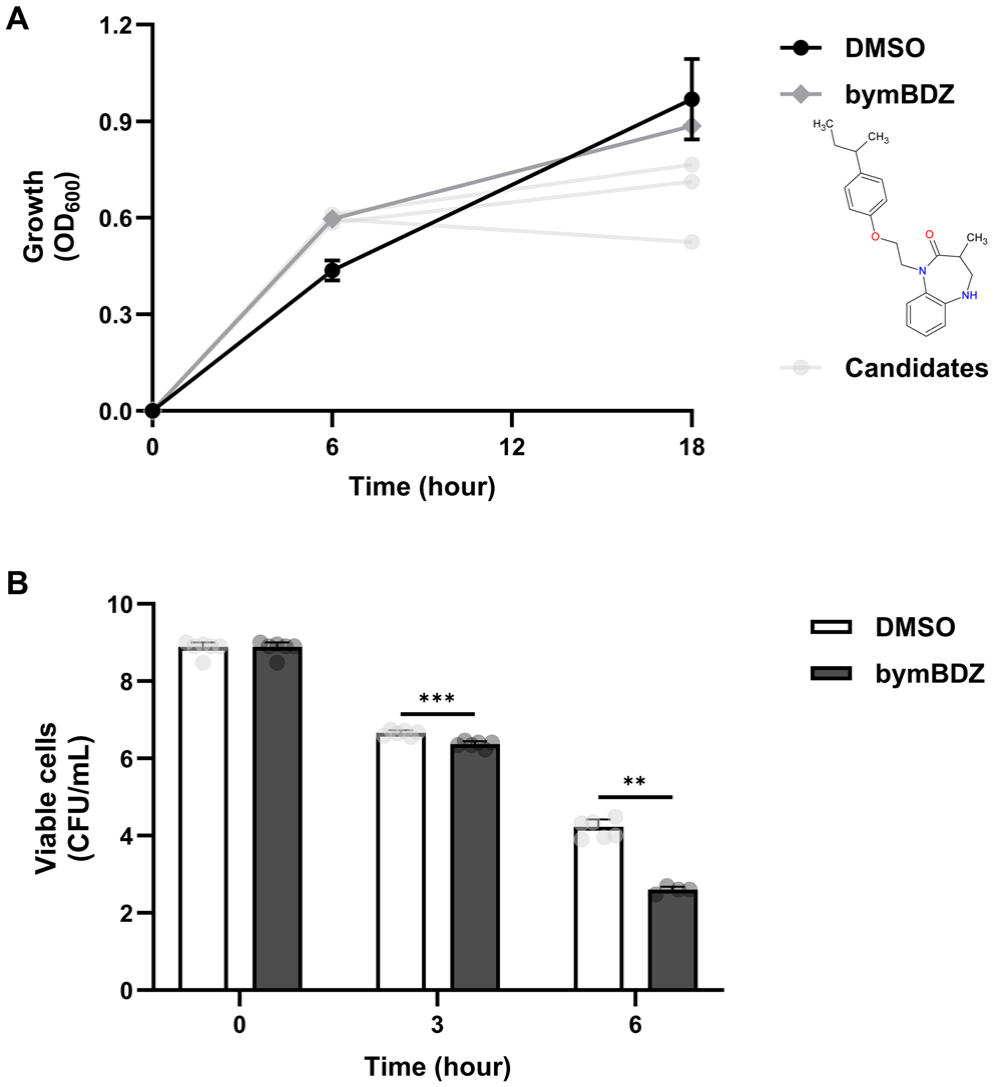
Screening of compounds that promote regrowth of *E. coli* persister cells. (A) Candidate compounds predicted to accelerate persister regrowth. Compounds were added in LB broth (100 μM), and *E. coli* BW25113 persister cells were inoculated. Turbidity (OD_600_) was measured at 0, 6 and 18 h, and all values were corrected by background. (B) Ampicillin time‐kill curves of *E. coli* cells treated with bymBDZ. bymBDZ (100 μM) was added to *E. coli* BW25113 cultures in LB medium containing ampicillin (100 μg/mL). DMSO was used as a vehicle. At 0, 3, 6 and 18 h, cultures were serially diluted with 0.85% NaCl, and 10 μL aliquots were spotted in triplicate onto LB agar plates for viable cell counting. Data represents the mean ± SD of two independent experiments. Statistical analysis was performed using two‐way ANOVA in GraphPad Prism 10. *p* ≤ 0.05 (*), *p* ≤ 0.01 (**) and *p* ≤ 0.001 (***) were considered statistically significant.

To identify chemicals that accelerate persister regrowth, a library of 7040 chemicals was screened using persister‐derived *E. coli* BW25113 cells generated by ampicillin treatment after rifampicin pretreatment. The chemicals were added individually to LB medium, and regrowth kinetics were monitored by measuring optical density at 600 nm (OD_600_) over 24 h. Chemicals that increased OD_600_ at 6 h relative to the DMSO control were selected as candidates. Four top hits consistently accelerated early regrowth (Figure 1A; Table 2). Notably, bymBDZ showed the strongest regrowth effect, increasing OD_600_ at 6 h to approximately 0.6 compared with approximately 0.4 for DMSO. By 18 h, culture density in the bymBDZ‐treated group reached levels comparable to the DMSO control, whereas several other hits displayed reduced endpoint OD_600_, indicating that these chemicals primarily influence regrowth timing rather than maximal growth yield (Figure 1A).

**TABLE 2:** Candidate compounds that accelerate persister cell regrowth.

Name	Structure
5‐[2‐(4‐butan‐2‐ylphenoxy)ethyl]‐3‐methyl‐2,3‐dihydro‐1H‐1,5‐benzodiazepin‐4‐one	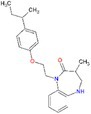
5‐cyclohexyl‐N‐[(1‐ethyl‐3,5‐dimethylpyrazol‐4‐yl)methyl]‐1,3,4‐oxadiazol‐2‐amine	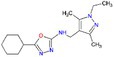
2‐[(4‐Tert‐butylphenyl)methyl]‐4‐(hydroxymethyl)‐5,6‐dimethylpyridazin‐3‐one	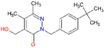
(7‐{[4‐(2‐Pyrimidinyl)‐1‐piperazinyl]sulfonyl}‐2H‐1,2,4‐benzothiadiazine 1,1‐dioxide)	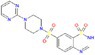

To test whether these hits potentiate antibiotic killing, *E. coli* cells were treated with ampicillin (100 μg/mL) in combination with each chemical (100 μM), and viable counts were determined (Figure 1B; Figure S1; Table S2A,B). Among the tested chemicals, bymBDZ most strongly enhanced ampicillin‐mediated killing, resulting in approximately 42‐fold lower survival at 6 h compared with ampicillin alone (Figure 1B; Table S2A). Together, these results identify bymBDZ as a chemical hit that accelerates persister regrowth and potentiates ampicillin killing.

### 3.2 bymBDZ Induces dcrB to Accelerate Regrowth of *E. coli* Persister Cells

#### 3.2.1 ASKA Overexpression Screening Identifies Candidate Factors

To identify proteins associated with bymBDZ‐driven acceleration of persister regrowth, we screened an *E. coli* ASKA overexpression library comprising 4267 proteins (Kitagawa et al. 2005). Persister cells from the pooled ASKA library were generated, then plated on M9/0.4% glucose containing bymBDZ (100 μM). Among approximately 377 colonies that formed, those appearing earliest or exhibiting the largest sizes were selected (Table S3). This screen identified nine candidate proteins (ArgT, Cdd, CheY, CspE, DcrB, PgsA, TrpS, YihA, YsgA).

**FIGURE 2:**
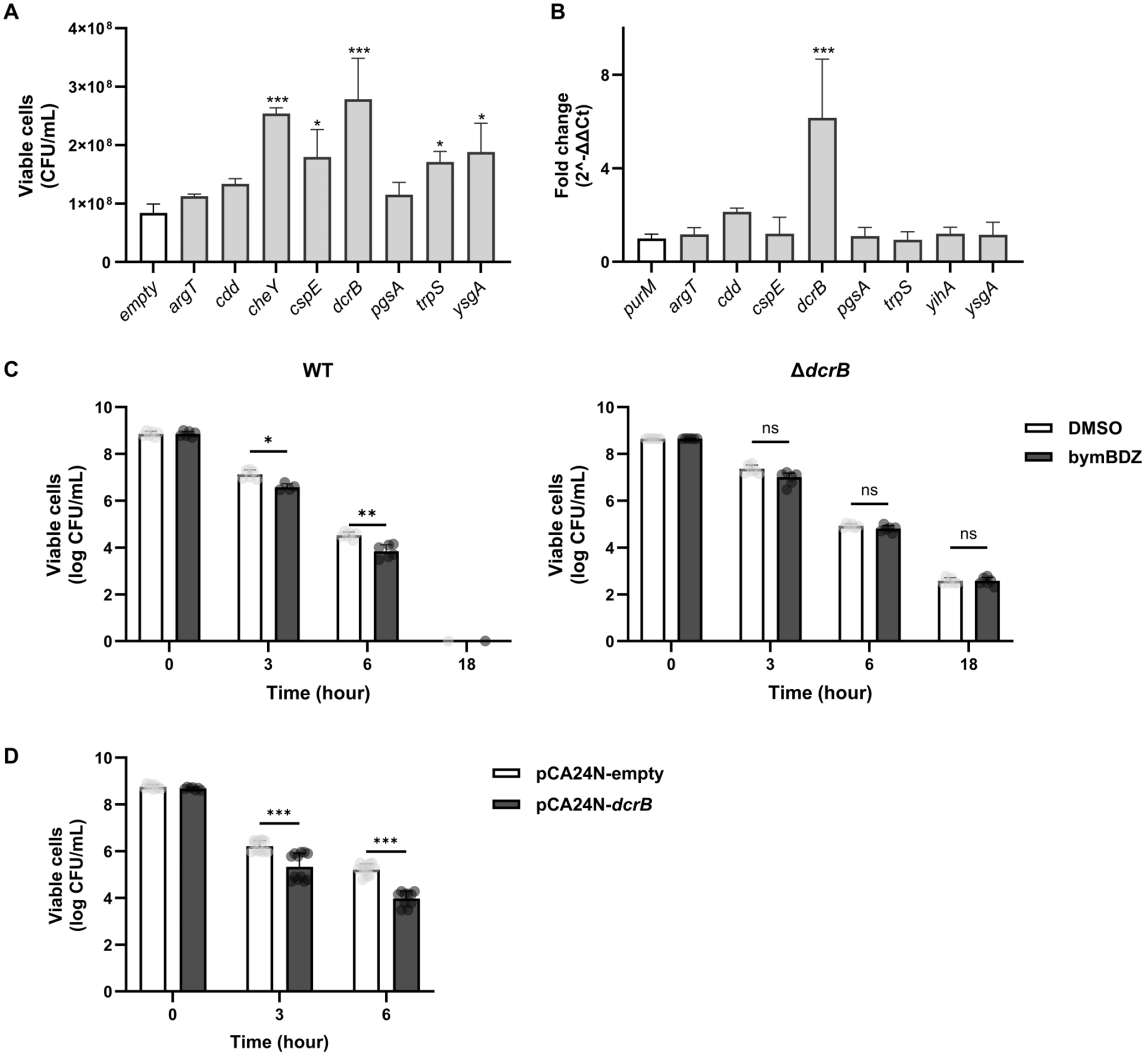
Identification of bymBDZ‐induced genes involved in *E. coli* persister cell resuscitation and regrowth. (A) Accelerated regrowth of persister cells by bymBDZ‐induced genes. Persister cells of *E. coli* carrying plasmids with selected genes in pCA24N were formed as previously described, and the diluent (10^−5^) was plated on M9 glucose (0.4%) agar. Viable cells of resuscitated persister cells were counted. Statistical significance was determined using Student's *t*‐test in GraphPad Prism. *p* ≤ 0.05 (*), *p* ≤ 0.01 (**) and *p* ≤ 0.001 (***) were considered statistically significant. (B) Gene expression analysis of bymBDZ‐induced genes. *E. coli* persister cells were exposed to M9/glucose (0.4%) containing DMSO or bymBDZ (100 μM) for resuscitation, and the total RNA was extracted. Expression of bymBDZ‐targeted candidate genes was analysed by quantitative RT‐PCR. Data were normalized to *purM* and are presented as fold changes relative to the DMSO‐treated control (mean ± SEM, *n* = 3). Statistical significance was determined using Student's *t*‐test in GraphPad Prism. *p* < 0.001. (C) Ampicillin time‐kill curves of Δ*dcrB* strain treated with bymBDZ. bymBDZ (100 μM) was added to cultures of *E. coli* BW25113 wild‐type and Δ*dcrB* in LB medium containing ampicillin (100 μg/mL). DMSO was used as a vehicle. At 0, 3, 6 and 18 h, cultures were serially diluted with 0.85% NaCl, and 10 μL aliquots were spotted in triplicate onto LB agar plates for viable cell counting. Data represents the mean ± SD of two independent experiments. Statistical analysis was performed using two‐way ANOVA in GraphPad Prism 10. *p* ≤ 0.05 (*), *p* ≤ 0.01 (**) and *p* ≤ 0.001 (***) were considered statistically significant. (D) Ampicillin time‐kill curve of *E. coli* pCA24N‐*dcrB*. *E. coli* BW25113/pCA24N‐*dcrB* cultures were treated with ampicillin (100 μg/mL) in LB media. At 0, 3 and 6 h, cultures were serially diluted with 0.85% NaCl, and 10 μL aliquots were spotted in triplicate onto LB agar plates for viable cell counting. Data represents the mean ± SD of two independent experiments. Statistical analysis was performed using two‐way ANOVA in GraphPad Prism 10. *p* ≤ 0.05 (*), *p* ≤ 0.01 (**) and *p* ≤ 0.001 (***) were considered statistically significant.

Individual overexpression of each candidate significantly enhanced regrowth, as shown by the increase in resuscitated persister cells at 18 h for CheY (3.02‐fold), CspE (2.14‐fold), DcrB (3.32‐fold), TrpS (2.04‐fold) and YsgA (2.24‐fold) compared to the empty plasmid control (Figure 2A; Table S4). Because CheY promotes persister regrowth through chemotaxis signalling (Bakali et al. 2007), we asked whether bymBDZ affects ribosome reactivation, a reported downstream step associated with CspE and TrpS‐linked recovery (Hu et al. 2015; Wood Whitney et al. 2021). Using *E. coli* MG1655 *rrnbP1*::GFP[ASV] as a ribosome reactivation reporter, bymBDZ‐treated persister cells showed a trend toward higher GFP‐positive fractions at 1 and 3 h versus DMSO controls, but differences were not statistically significant (Figure S2; Table S5). These results suggest that bymBDZ does not robustly trigger ribosome reactivation independently under these conditions and that translational restart likely may occur downstream of upstream regrowth‐promoting events.

#### 3.2.2 *dcrB* Is Induced by bymBDZ and Is Necessary and Sufficient for Potentiating Ampicillin Killing

Transcript levels of candidate genes were quantified by qRT‐PCR in persister cells resuming growth in M9/0.4% glucose with bymBDZ. Among the tested candidates, *dcrB* showed the strongest response, with approximately six‐fold induction (Figure 2B). *dcrB* deletion reduced ampicillin susceptibility and abolished the sensitizing effect of bymBDZ (Figure 2C; Figure S3A; Table S6A,B). Unlike wild‐type exponential‐phase cells treated with the bymBDZ–ampicillin combination, Δ*dcrB* cells showed no enhanced killing at 3 h (−2.27‐fold) or 6 h (−1.32‐fold), with survival comparable to controls up to 18 h (Figure 2C; Table S6A). Whereas the other candidates showed little or no effect (Figure S3C; Table S7B), overexpression of *dcrB* alone resulted in approximately 4.5‐fold and 16.3‐fold greater ampicillin killing at 3 and 6 h, respectively, versus empty‐plasmid control (Figure 2D; Figure S3B; Table S7A), closely resembling the bymBDZ–ampicillin pattern (Figure 2C,D; Figure S3A,B; Tables S6, S7A). Collectively, these results demonstrate that bymBDZ‐induced *dcrB* upregulation is both necessary and sufficient for enhanced ampicillin killing.

### 3.3 DcrB Represses Envelope Genes and Enhances Intracellular Small‐Molecule Exposure

**FIGURE 3:**
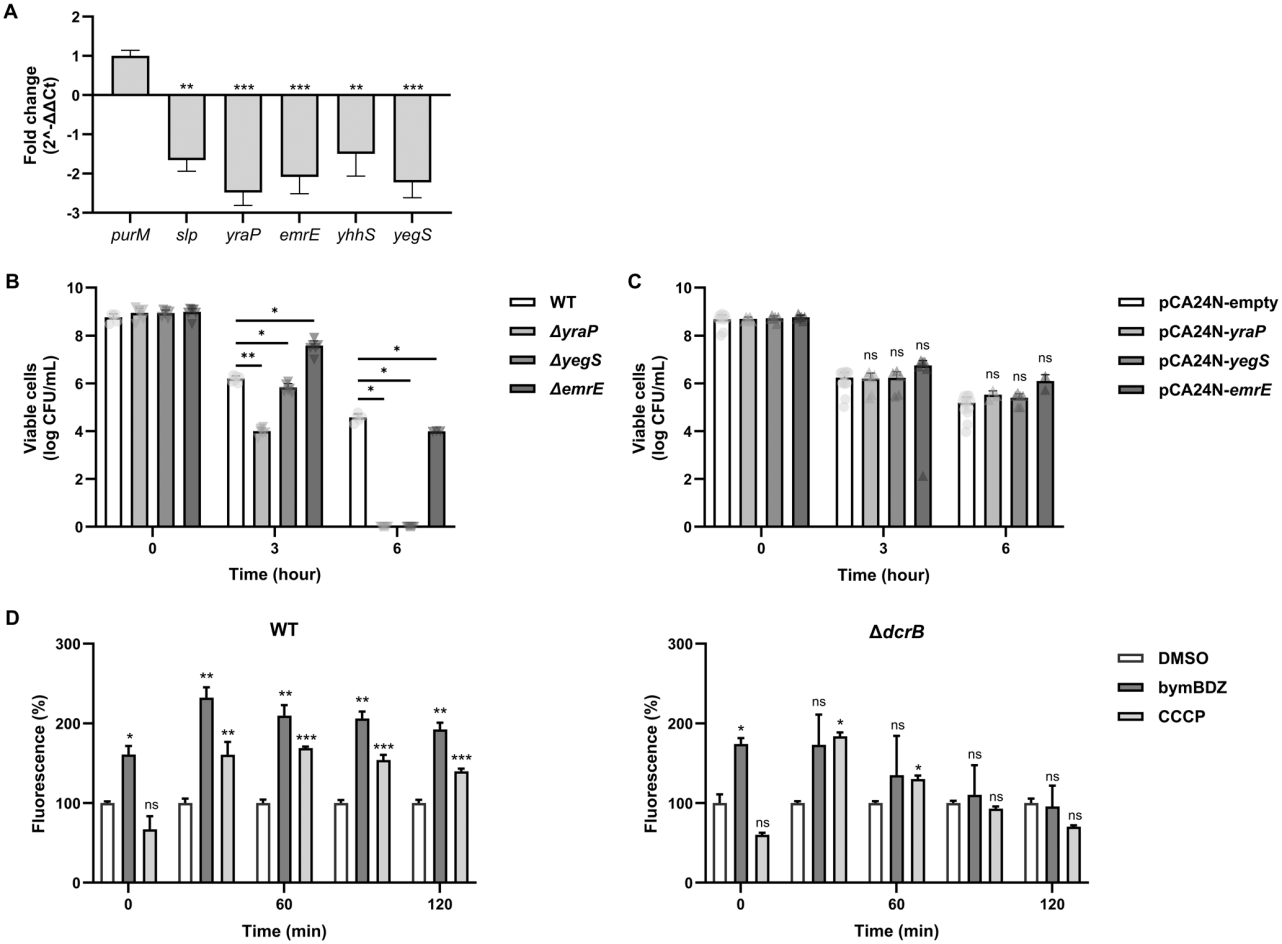
Role of *dcrB* and related genes in antibiotic killing and intracellular accumulation. (A) mRNA expression of *dcrB* related genes. *E. coli* cells carrying pCA24N‐empty or pCA24N‐*dcrB* were treated with IPTG for plasmid overproduction, and the total RNA was extracted. Expression of *dcrB*‐related genes was analysed by quantitative RT‐PCR. Data were normalized to *purM* and are presented as mean ± SD. Statistical analysis was performed using Students' *t*‐test in GraphPad Prism 10. *p* ≤ 0.05 (*), *p* ≤ 0.01 (**) and *p* ≤ 0.001 (***) were considered statistically significant. (B) Time‐kill curve of *dcrB‐*related genes. *E. coli* strains with lacking or overproducing *dcrB‐*related genes were treated with ampicillin (100 μg/mL) in LB medium. At 0, 3 and 6 h, cultures were serially diluted with 0.85% NaCl, and 10 μL aliquots were spotted in triplicate onto LB agar plates for viable cell counting. Data represents the mean ± SD of two independent experiments. Statistical analysis was performed using two‐way ANOVA in GraphPad Prism 10. *p* ≤ 0.05 (*), *p* ≤ 0.01 (**) and *p* ≤ 0.001 (***) were considered statistically significant. (C) Effect of bymBDZ on intracellular Hoechst accumulation. Wild‐type or Δ*dcrB* cells turbidity at 600 nm of 0.3 were resuspended in PBS and treated with DMSO or bymBDZ (100 μM). Hoechst 33342 was added to a final concentration of 2.5 μM, and fluorescence was monitored from the top of the wells using excitation/emission wavelengths of 360/460 nm over 9 cycles at 15‐min intervals. Data represents the mean ± SD of two independent experiments. Statistical analysis was performed using two‐way ANOVA in GraphPad Prism 10. *p* ≤ 0.05 (*), *p* ≤ 0.01 (**) and *p* ≤ 0.001 (***) were considered statistically significant.

To better understand how DcrB influences the bymBDZ‐driven phenotype that enhances ampicillin killing, we examined the expression of the five envelope‐related genes (slp, *yraP*, *emrE*, *yhhS*, *yegS*) previously linked to DcrB (Pushpker et al. 2022; Szklarczyk et al. 2023). qRT–PCR analysis showed that *dcrB* overexpression reduced the transcript levels of all five genes, with the most pronounced decreases observed for *yraP*, *yegS* and *emrE* (approximately two‐fold or greater) (Figure 3A). These genes have been implicated in envelope homeostasis and membrane transport in Gram‐negative bacteria (Yerushalmi et al. 1995; Bakali et al. 2007; Bryant et al. 2020).

We next evaluated whether repression of these DcrB‐responsive genes contributes to antibiotic‐mediated killing. Under ampicillin treatment, Δ*yraP*, Δ*yegS* and Δ*emrE* mutants exhibited increased killing compared to the wild‐type strain. At 3 h, Δ*yraP* and Δ*yegS* showed 158‐fold and 2.3‐fold lower survival, respectively, and no viable cells were detected for either mutant at 6 h whereas wild‐type still retained survivors. The Δ*emrE* mutant also displayed enhanced killing, with an approximately 3.9‐fold lower survival at 6 h (Figure 3B; Figure S4; Table S8A). In contrast, overexpression of the corresponding genes had little or no effect on ampicillin killing (Figure 3B; Figure S4; Table S8B). Among the genes downregulated upon *dcrB* overexpression, *emrE* encodes a small multidrug transporter, and *yhhS* is annotated as an inner membrane transport‐associated protein (Koita and Rao 2012; Bryant et al. 2020). Together with the reported envelope‐ and stress‐associated roles of *yraP*, *yegS* and *slp*, this expression pattern suggested that *dcrB* induction by bymBDZ might impact envelope‐associated transport and or intracellular retention during early regrowth. We therefore tested whether bymBDZ increases intracellular exposure to small molecules by quantifying intracellular accumulation of the DNA‐binding fluorescent dye Hoechst 33342 (H33342) as a proxy for the balance between dye influx or permeability and energy‐dependent export.

In wild‐type cells, bymBDZ increased H33342 accumulation relative to the DMSO control, reaching ~2‐fold at 30 min and remaining elevated (~1.9–2.3‐fold) throughout the assay (Figure 3C). CCCP (carbonyl cyanide‐m‐chlorophenylhydrazone) similarly increased H33342 accumulation, consistent with reduced energy‐dependent export. In the Δ*dcrB* mutant, bymBDZ still increased H33342 accumulation compared to DMSO, but the response was reduced in magnitude and less sustained, with a lower peak (~2.1‐fold) and a faster return toward baseline at later time points. CCCP‐induced accumulation also appeared less sustained in Δ*dcrB*. Together, these results support the notion that bymBDZ increases intracellular dye exposure through both DcrB‐independent and DcrB‐dependent processes, with DcrB contributing to a maximal, sustained accumulation profile (Figure 3C).

### 3.4 MdtL Is Required for bymBDZ‐Induced *dcrB* Expression and Ampicillin Killing

**FIGURE 4:**
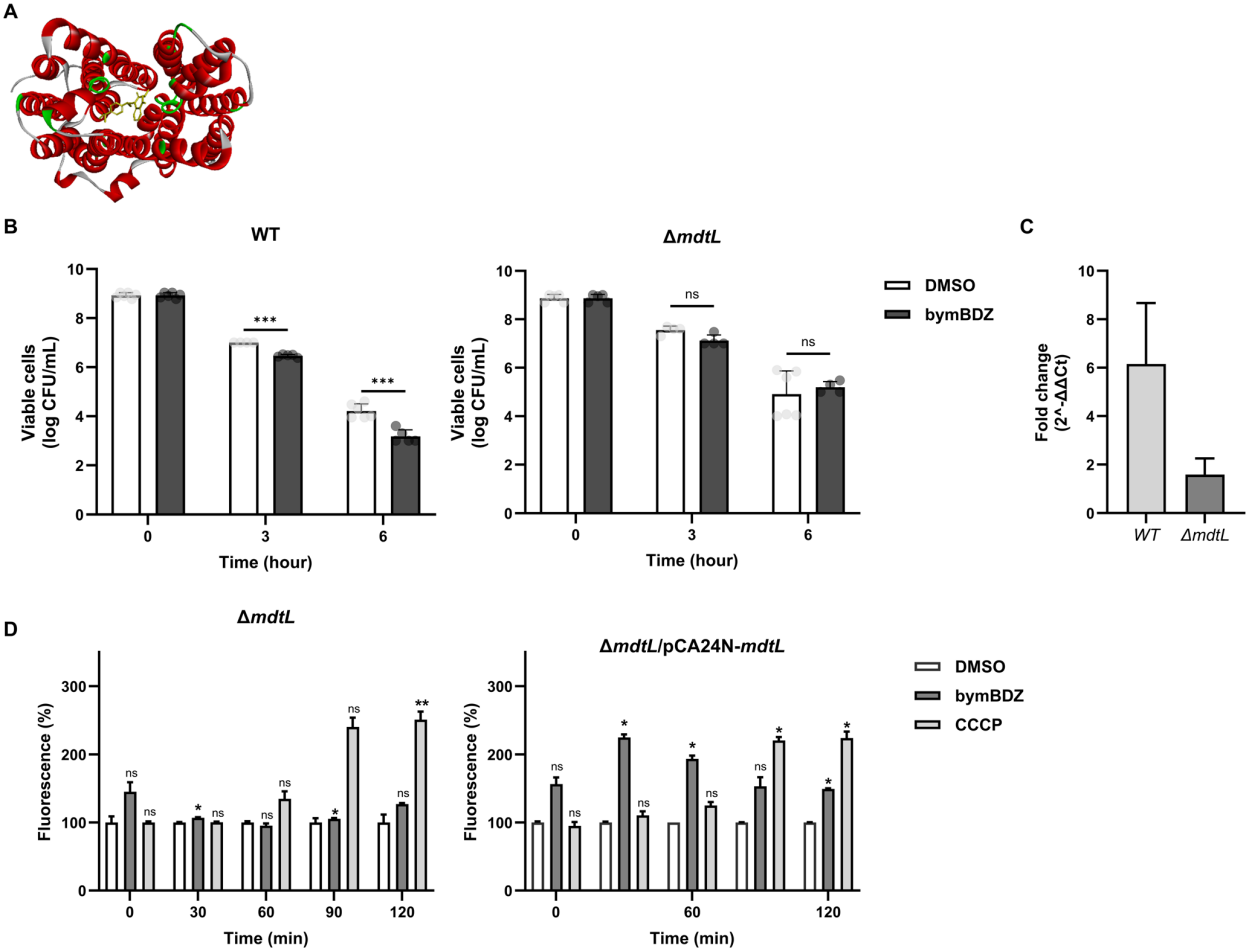
Docking analysis of bymBDZ with the transporter MdtL and functional validation. (A) Predicted binding mode of bymBDZ to the target protein MdtL. Docking poses and binding affinity were calculated using the AutoDock Vina v1.2.7. The structure of MdtL was obtained from AlphaFold predictions. Binding pockets were identified using POCOSA, and docking results were visualized using Discovery Studio. (B) Ampicillin time–kill curves of wild‐type and Δ*mdtL* strains treated with bymBDZ. *E. coli* BW25113 wild‐type or Δ*mdtL* cells were treated with ampicillin (100 μg/mL) and bymBDZ (100 μM) in LB medium. At 0, 3 and 6 h, cultures were serially diluted with 0.85% NaCl, and 10 μL aliquots were spotted in triplicate onto LB agar plates for viable cell counting. Data represents the mean ± SD of two independent experiments. Statistical analysis was performed using two‐way ANOVA in GraphPad Prism 10. *p* ≤ 0.05 (*), *p* ≤ 0.01 (**) and *p* ≤ 0.001 (***) were considered statistically significant. (C) Expression of *dcrB* in the Δ*mdtL* strain. Persister cells of the Δ*mdtL* strain were exposed to M9/glucose (0.4%) containing DMSO or bymBDZ (100 μM) for 30 min to resuscitate, after which total RNA was extracted. Expression of *dcrB* was analysed by quantitative RT‐PCR. Data were normalized to *purM* and are presented as mean ± SD. (D) Effect of bymBDZ on MdtL‐mediated intracellular Hoechst accumulation. Wild‐type, Δ*mdtL* and Δ*mdtL*/pCA24N‐*mdtL* cells turbidity at 600 nm of 0.3 were resuspended in PBS and treated with DMSO or bymBDZ (100 μM). Hoechst 33342 was added to a final concentration of 2.5 μM, and fluorescence was monitored from the top of the wells using excitation/emission wavelengths of 360/460 nm over 9 cycles at 15‐min intervals. Data represents the mean ± SD of two independent experiments. Statistical analysis was performed using two‐way ANOVA in GraphPad Prism 10. *p* ≤ 0.05 (*), *p* ≤ 0.01 (**) and *p* ≤ 0.001 (***) were considered statistically significant.

To identify membrane components that may act upstream of DcrB in the bymBDZ response, we performed in silico docking analysis using AutoDock Vina (Trott and Olson 2010). bymBDZ showed a modest predicted affinity for DcrB (−6.089 kcal/mol), whereas higher‐scoring interactions were predicted for several membrane proteins, including EmrY (−8.859 kcal/mol), MdtG (−8.396 kcal/mol), Tsx (−8.379 kcal/mol), MdtL (−8.249 kcal/mol) and GlpT (−8.015 kcal/mol) (Figure 4A; Figure S5). Because docking provides hypothesis‐generating rather than definitive functional evidence, we next tested whether these candidate proteins contribute to the bymBDZ‐dependent phenotype by examining the corresponding knockout strains. Among the tested mutants, Δ*mdtL* uniquely failed to show enhanced killing upon bymBDZ–ampicillin treatment at 3 or 6 h, unlike the wild‐type and the other mutants (Figure 4B; Figure S5; Table S9). Consistent with this phenotype, bymBDZ‐dependent induction of *dcrB* was abolished in the Δ*mdtL* background (Figure 4C). In addition, Δ*mdtL* cells did not show increased H33342 accumulation upon bymBDZ treatment relative to the DMSO control over the assay period. By contrast, complementation of Δ*mdtL* with pCA24N‐*mdtL* restored the bymBDZ‐dependent increase in H33342 accumulation, reaching ~2.5‐fold at 30 min and remaining above the DMSO control throughout the assay (Figure 4D; Figure S6).

Together, these results support that MdtL is required for bymBDZ‐induced *dcrB* expression and for potentiation of ampicillin‐mediated killing, placing MdtL upstream of the DcrB‐dependent sensitization pathway.

### 3.5 bymBDZ Potentiates Antibiotic Killing in Pathogenic EHEC and Across Selected Antibiotic Classes

**FIGURE 5:**
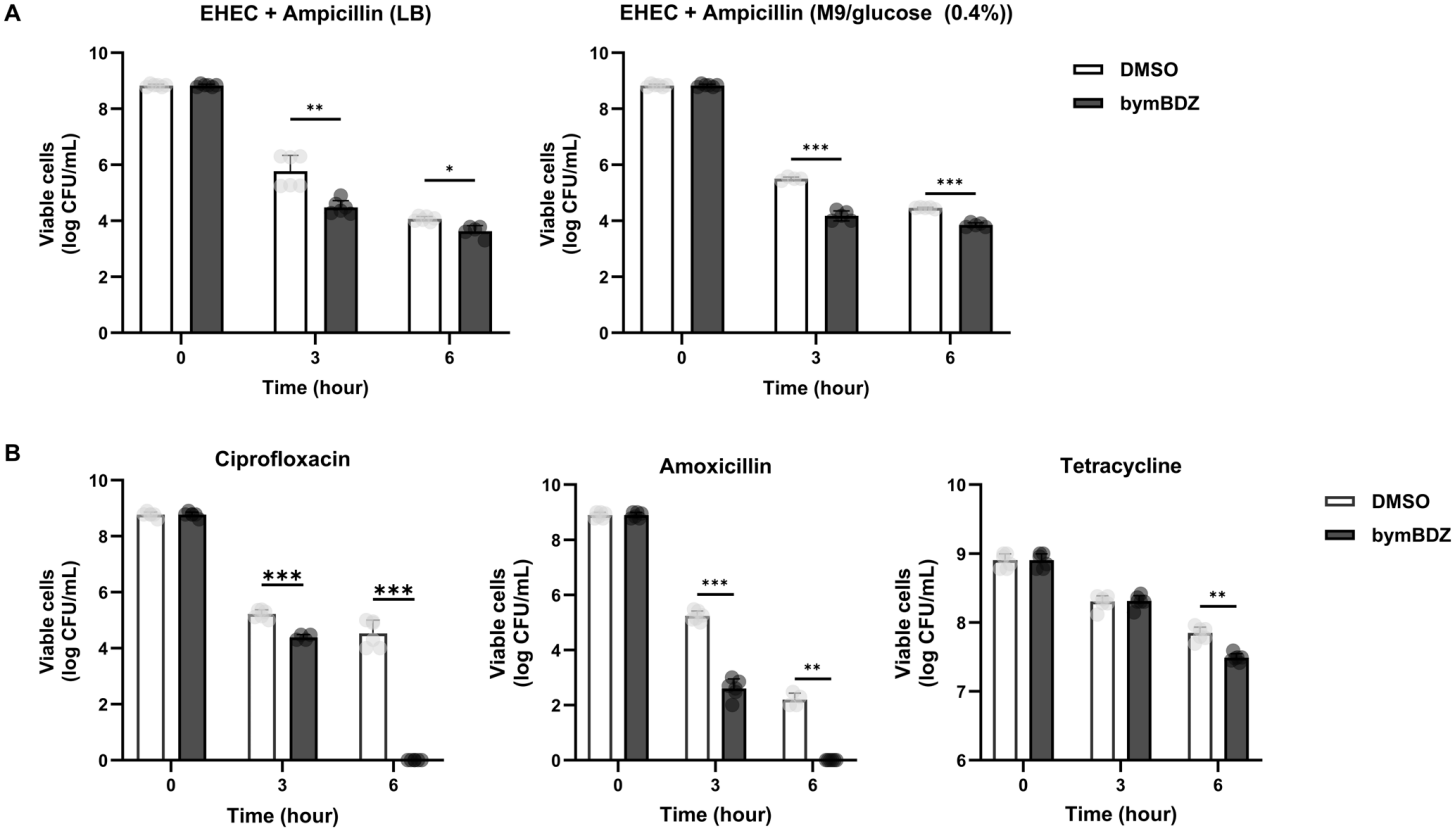
Application to *E. coli* O157:H7 ATCC 43889 and various antibiotic combinations. (A) Ampicillin kill curve of *E. coli* O157:H7 (ATCC 43889). *E. coli* O157:H7 (ATCC 43889) cells were treated with ampicillin (100 μg/mL) and bymBDZ (100 μM) in LB or M9/glucose (0.4%) media. At 0, 3, 6 and 18 h, cultures were serially diluted with 0.85% NaCl, and 10 μL aliquots were spotted in triplicate onto LB agar plates for viable cell counting. Data represents the mean ± SD of two independent experiments. Statistical analysis was performed using two‐way ANOVA in GraphPad Prism 10. *p* ≤ 0.05 (*), *p* ≤ 0.01 (**) and *p* ≤ 0.001 (***) were considered statistically significant. (B) bymBDZ in combination antibiotics kill curves of *E. coli* BW25113 cells. *E. coli* BW25113 cells were treated with ciprofloxacin (5 μg/mL), amoxicillin (100 μg/mL), tetracycline (100 μg/mL) and bymBDZ (100 μM) in LB medium. At 0, 3, 6 and 18 h, cultures were serially diluted with 0.85% NaCl, and 10 μL aliquots were spotted in triplicate onto LB agar plates for viable cell counting. Data represents the mean ± SD of two independent experiments. Statistical analysis was performed using two‐way ANOVA in GraphPad Prism 10. *p* ≤ 0.05 (*), *p* ≤ 0.01 (**) and *p* ≤ 0.001 (***) were considered significant.

To assess the translational potential of bymBDZ, we tested its activity in enterohemorrhagic *E. coli* O157:H7 (ATCC 43889) and in combination with antibiotics with distinct mechanisms of action. In EHEC, bymBDZ potentiated ampicillin killing in both media. In LB, the combination produced ~5.4‐fold greater killing than ampicillin alone, whereas in M9/0.4% glucose the potentiation was stronger (~21.2‐fold), with maximal effects observed at 3 h (Figure 5A; Table S10).

We next asked whether potentiation extends across antibiotic classes (Figure S7A–D; Table S11A–D). bymBDZ enhanced ciprofloxacin activity, reducing viable cells by ~7‐fold more than ciprofloxacin alone at 3 h in LB and eliminating detectable survivors by 6 h, while ciprofloxacin alone still left survivors at this time point (Figure 5B; Table S11A). In M9/0.4% glucose, the combination yielded ~2‐fold and ~36‐fold greater reductions in viability at 3 h and 6 h, respectively, compared with ciprofloxacin alone. Survivors became undetectable by 18 h with bymBDZ co‐treatment, whereas ciprofloxacin alone still left survivors (Figure S7A; Table S11A).

bymBDZ also potentiated amoxicillin killing. In LB, the combination produced ~370‐fold greater killing than amoxicillin alone at 3 h (Figure 5B; Table S11B) and in M9/0.4% glucose the effect was ~5.8‐fold at 3 h (Figure S7B; Table S11B). In both media, no viable cells were detected from 6 h onward under the combination condition, whereas amoxicillin alone still left detectable survivors. For tetracycline, bymBDZ increased killing at 6 h (~2‐fold in LB and ~12‐fold in M9/0.4% glucose relative to tetracycline alone), although longer incubation resulted in survival levels similar to tetracycline alone (Figure 5B; Figure S7C; Table S11C).

Collectively, these results show that bymBDZ‐mediated potentiation is observed in a pathogenic *E. coli* background and extends to multiple antibiotic classes, with the most pronounced effects under conditions and time windows where survivors are otherwise retained during early killing assays.

## 4 Discussion

Persister cells are transiently tolerant subpopulations that survive antibiotic exposure without acquiring heritable resistance, rendering them difficult to eradicate with conventional antibiotics (Shah et al. 2006; Harms et al. 2016; Fisher et al. 2017; Song and Wood 2021; Arvaniti and Skandamis 2022). The clinical significance of persister cells is underscored by their proposed role in the recurrence of chronic and relapsing bacterial infections, where tolerant subpopulations survive antibiotic treatment and repopulate the infection site upon drug removal, contributing to treatment failure (Harms et al. 2016; Fisher et al. 2017; Fang and Allison 2023). Current anti‐persister strategies include direct killing, prevention of persister formation, and restoration of antibiotic susceptibility by manipulating persister physiology to enhance combination therapies (Defraine et al. 2018). Among these approaches, accelerating the transition from dormancy to active growth is particularly attractive because most antibiotics are most effective when essential biosynthetic processes are active (Forge and Schacht 2000; Schneider and Sahl 2010; Arenz and Wilson 2016; Pham et al. 2019).

In this study, we identify bymBDZ as a chemical hit that shortens the lag to growth resumption in persister‐derived *E. coli* and potentiates antibiotic killing (Figure 1; Table S2A). In primary regrowth assays, bymBDZ promoted earlier regrowth while endpoint densities converged with controls, indicating a timing‐driven effect rather than increased maximal growth yield (Figure 1A). This is notable because persister cells can exhibit prolonged lag times relative to actively growing cells, and single‐cell observations indicate that awakening kinetics are constrained by ribosome content, with ribosome‐poor persisters recovering more slowly (Kim et al. 2018). Shortening this lag may increase the fraction of cells that reach an antibiotic‐susceptible state within a defined treatment window. In line with this view, bymBDZ enhanced ampicillin‐mediated killing during early time windows when survivors are typically retained (Figure 1B; Figure S1B; Table S2A).

Unlike previously described persister wakers identified through direct resuscitation screens, such as BPOET (Song and Wood 2020a, 2020b), bymBDZ does not primarily act as an awakening trigger. Instead, it shortens the lag and accelerates early regrowth after growth resumption has begun. This distinction is important because resuscitation is asynchronous and heterogeneous across persister‐derived cells (Yu et al. 2019; Fang and Allison 2023). In this framework, the therapeutic benefit of bymBDZ arises from advancing early regrowth so that a larger fraction of cells enters an antibiotic‐susceptible, growth‐dependent physiological state within a defined treatment window (Figure 1; Table S2A).

To identify determinants linking bymBDZ to accelerated recovery, we performed an ASKA overexpression screen as a hypothesis‐generating approach (Kitagawa et al. 2005). This screen nominated candidate factors associated with enhanced regrowth under bymBDZ exposure (Table S3), with follow‐up testing confirming reproducible regrowth enhancement for CheY, CspE, DcrB, TrpS and YsgA (Figure 2A; Table S4). These candidates map onto processes previously implicated in persister physiology and awakening, including chemotaxis‐linked nutrient sensing and early recovery programs supporting translational restart (Yamasaki et al. 2020; Song et al. 2021). Notably, genetic loss‐ and gain‐of‐function analyses converged on *dcrB* as a central causal node in the bymBDZ response (Figure 2C,D; Figure S3A,B; Tables S6 and S7A).

DcrB induction during regrowth provides a mechanistic entry point into bymBDZ‐driven physiological remodelling. Among screened candidates, bymBDZ induced *dcrB* transcripts most prominently (Figure 2B). *dcrB* deletion abolished bymBDZ‐mediated antibiotic potentiation, whereas *dcrB* overexpression alone recapitulated the enhanced killing phenotype (Figure 2C,D; Figure S3A,B; Tables S6 and S7A), demonstrating that *dcrB*‐dependent processes are both necessary and sufficient for sensitization.

Consistent with prior reports implicating DcrB in envelope‐associated stress responses (Likhacheva et al. 1990; Samsonov et al. 2002; Pushpker et al. 2022; Szklarczyk et al. 2023), qRT‐PCR showed that *dcrB* overexpression represses *yraP* (*dolP*), *yegS* and *emrE* (Figure 3A). These genes are linked to envelope homeostasis and transport, and EmrE has been reported to be phenotypically associated with DcrB under osmotic stress (Yerushalmi et al. 1995; Bakali et al. 2007; Bryant et al. 2020; Pushpker et al. 2022). Functionally, deletions of *yraP*, *yegS* and *emrE* enhanced ampicillin‐mediated killing and reduced survivor persistence during early assays, with complementation restoring wild‐type survival (Figure 3B; Figure S4; Table S8). Together, these genetic phenotypes support a model wherein DcrB‐linked envelope remodelling shifts cells toward an antibiotic‐susceptible state during early regrowth, potentially by altering envelope homeostasis and the net intracellular exposure to small molecules (Figure 3A,B; Figure S4; Table S8).

Previous work indicates that the role of efflux in persistence is highly context dependent. Persister cells have been reported to maintain active efflux that limits intracellular antibiotic accumulation (Pu et al. 2016), whereas other studies suggest that efflux can be diminished in dormancy, allowing antibiotics such as minocycline to accumulate and drive rapid killing upon resuscitation (Roy et al. 2021). Together, these findings suggest that envelope‐linked transport states can shape intracellular drug exposure and thereby influence both persister survival and recovery outcomes. We therefore hypothesized that bymBDZ enhances killing during early regrowth by shifting the net balance of influx and export, leading to increased intracellular exposure to small molecules.

To test whether bymBDZ increases net intracellular exposure during early regrowth, we quantified Hoechst 33342 accumulation as a proxy for influx/export balance (Coldham et al. 2010). bymBDZ markedly increased intracellular dye accumulation relative to controls, similar to the proton motive force uncoupler CCCP (Mini et al. 2022; Li et al. 2023) (Figure 3C). PMF has been recognized as a critical determinant of antibiotic tolerance, as its active maintenance supports efflux activity and other energy‐dependent survival functions in persisters (Wan et al. 2024). While CCCP sensitizes bacteria by directly dissipating PMF, the bymBDZ‐driven accumulation profile differed notably from that of CCCP. Most notably, bymBDZ‐induced accumulation was abolished in the Δ*mdtL* background (Figure 4D; Figure S6), whereas CCCP‐mediated accumulation was not, suggesting that bymBDZ may act, at least in part, through a transporter‐specific mechanism. Whether bymBDZ additionally modulates PMF remains to be directly determined. In the Δ*dcrB* background, the bymBDZ‐driven accumulation profile was attenuated and less sustained (Figure 3C), indicating that DcrB contributes to maximal, sustained intracellular exposure alongside DcrB‐independent effects.

To examine whether bymBDZ increases ampicillin effectiveness directly, we determined MIC_100_ and MIC_50_ values for ampicillin in wild‐type and Δ*dcrB* strains in the presence or absence of bymBDZ (Table S12). bymBDZ reduced MIC_100_ by 2‐fold in both backgrounds (8 μg/mL → 4 μg/mL), with MIC_50_ values similarly reduced in both strains (wild‐type: 3.72 → 2.37 μg/mL; Δ*dcrB*: 3.35 → 2.34 μg/mL), confirming that bymBDZ modestly increases ampicillin sensitivity. This modest reduction is consistent with our Hoechst 33342 accumulation data showing that bymBDZ increased H33342 accumulation even in the Δ*dcrB* background, albeit with reduced magnitude and faster return toward baseline (Figure 3C), suggesting that partial, DcrB‐independent intracellular accumulation may contribute to the observed MIC shifts. Nonetheless, despite equivalent MIC reductions in both strain backgrounds, bymBDZ‐mediated killing potentiation was completely absent in the Δ*dcrB* mutant, indicating that the dominant killing effect cannot be attributed to direct ampicillin sensitization alone and instead requires an intact MdtL–DcrB axis. While we cannot fully exclude the possibility that some component of the killing enhancement reflects direct sensitization, the complete DcrB‐dependence of killing potentiation and the magnitude of the effect—up to 42‐fold with ampicillin and 370‐fold with amoxicillin—are more consistent with a primary mechanism operating through MdtL–DcrB‐dependent envelope remodelling than through direct modulation of ampicillin effectiveness.

We next identified membrane components acting upstream of DcrB. In silico docking nominated candidate transporters (Figure 4A; Figure S5), but genetic testing revealed that only Δ*mdtL* uniquely abolished bymBDZ‐mediated phenotypes (Figure 4B; Figure S5; Table S9). The Δ*mdtL* mutant failed to show bimBDZ‐dependent killing potentiation, *dcrB* induction or Hoechst accumulation, all of which were restored by complementation (Figure 4B–D; Figure S6; Table S9). These results establish an MdtL→DcrB signalling axis driving envelope remodelling and increased intracellular exposure during early regrowth (Figure 4B–D; Figure S6; Table S9).

Because growth resumption requires translational restart, we evaluated ribosome reactivation using a *rrnBP1* reporter. Under our conditions, bymBDZ produced only a modest, non‐significant trend in reporter activation (Figure S2; Table S5), suggesting translational restart is not a primary bymBDZ target. Instead, our data support a model wherein envelope‐associated transport remodelling and increased intracellular exposure precede and facilitate downstream translational reactivation (Figures 3 and 4; Figures S2, S6; Tables S5, S8, and S9).

The potentiating activity of bymBDZ extended to pathogenic enterohemorrhagic *E. coli* O157:H7 (Figure 5A; Table S10) and multiple antibiotic classes (Figure 5B; Figure S7; Table S11). Potentiation magnitude varied across media and antibiotics, consistent with context‐dependent benefits of shortened regrowth lag. Notably, the strongest effects occurred in conditions and time windows where survivors are typically retained (Figure 5; Tables S10 and S11).

Our data support a model for potentiation. bymBDZ engages an MdtL‐ and DcrB‐dependent envelope remodelling program that increases net intracellular exposure during early regrowth (Figures 3C and 4D; Figure S6). This transport shift is accompanied by a shortened lag and accelerated early regrowth, thereby increasing the fraction of persister‐derived cells that enter a growth‐dependent, antibiotic‐susceptible state within the treatment window (Figure 1A). Together, these changes reinforce antibiotic killing during early regrowth.

In summary, bymBDZ activates an MdtL→DcrB axis that remodels envelope‐associated transport and increases net intracellular exposure, which is accompanied by earlier regrowth and potentiated antibiotic killing of persister‐derived cells. Although bymBDZ modestly reduced ampicillin MIC₁₀₀ and MIC₅₀ by approximately 2‐fold in both wild‐type and Δ*dcrB* backgrounds (Table S12), consistent with partial DcrB‐independent intracellular accumulation (Figure 3C), the complete absence of killing potentiation in the Δ*dcrB* mutant despite equivalent MIC reductions suggests that this modest sensitization is unlikely to be the primary driver of the dominant MdtL–DcrB‐dependent killing potentiation (Figure 2C,D; Figure S3A,B; Tables S6 and S7A). Future work should determine whether the increased intracellular exposure reflects altered influx, reduced export, or both, and establish whether MdtL is a direct target of bymBDZ. The relative contributions of the DcrB‐dependent and DcrB‐independent components of bymBDZ activity also remain to be defined. Direct quantification of PMF using membrane potential‐sensitive probes such as DiOC_2_(3) would further clarify whether bymBDZ modulates the MdtL–DcrB axis independently of PMF dissipation, thereby strengthening the mechanistic distinction from classical PMF‐disrupting anti‐persister strategies.

## Author Contributions

**Hyein Kim:** investigation, data curation. **Sooyeon Song:** conceptualization, methodology, funding acquisition, supervision, formal analysis, writing – original draft, writing – review and editing, resources, project administration, investigation. **Garin Park:** methodology, software, investigation, validation, formal analysis, visualization, data curation, writing – review and editing, writing – original draft.

## Funding

This work was supported by National Research Foundation of Korea (NRF‐2020R1F1A1072397, RS‐2023‐00210305).

## Conflicts of Interest

The authors declare no conflicts of interest.

## Supporting information


**Table S1:** Primers used in this study.
**Table S2:** Ampicillin time kill curves with the candidate compounds. *E. coli* BW25113 cells were treated with ampicillin (100 μg/mL) and bymBDZ (100 μM) in LB or M9/glucose (0.4%). At 0, 3, 6 and 18 h, cultures were serially diluted with 0.85% NaCl, and 10 μL aliquots were spotted in triplicate onto LB agar plates for viable cell counting. Data represents the mean ± SD of two independent experiments. Statistical analysis was performed using two‐way ANOVA in GraphPad Prism 10. *p* ≤ 0.05 (*), *p* ≤ 0.01 (**) and *p* ≤ 0.001 (***) were considered statistically significant. Fold‐change values below 0.5 were transformed and reported as −1/fold change to indicate downregulation. Data for Figure 1B and Figure S1B are provided in (A) and data for Figure S1A is provided in (B).
**Table S3:** Viable cell counts of pooled ASKA persister cells on M9 glucose (0.4%) agar with bymBDZ (100 μM). All ASKA clones (GFP‐) were combined, grown together to a turbidity of 2 at 600 nm LB media, and the plasmids were isolated. The pooled ASKA plasmids were electroporated into *E. coli* BW25113 component cells, and the cells were cultured to exponential phase. The Persister cells of pooled ASKA strains were generated by sequential rifampicin (100 μg/mL) and ampicillin (100 μg/mL) treatment, washed twice with 0.85% NaCl, and resuscitated in M9/glucose (0.4%) medium containing bymBDZ. Fold‐change value below 0.5 were transformed and reported as −1/fold change to indicate downregulation.
**Table S4:** Resuscitation of persister cells by bymBDZ‐induced genes. Persister cells of *E. coli* carrying plasmids with selected genes were formed by treatment of rifampicin and ampicillin, washed twice with 0.85% NaCl, diluted to 10^5^, and plated on M9/glucose (0.4%) agar. Viable cells of persister cells resuscitated on M9/glucose (0.4%) agar was counted. Statistical significance was determined using Student's *t*‐test in GraphPad Prism. *p* ≤ 0.05 (*), *p* ≤ 0.01 (**) and *p* ≤ 0.001 (***) were considered statistically significant. All underlying data are consistent with the distributions shown in Figure 2A.
**Table S5:** Ribosome activation of *E. coli* by bymBDZ. The persister cells *E. coli* MG1655 *rrnbP1*::GFP[ASV] were generated as described previously and resuscitated on M9/glucose (0.4%) agarose gel pad containing DMSO or bymBDZ (100 μM). The green fluorescent protein (GFP) signal of the resuscitating persisters was monitored. Representative images are shown in Figure S2.
**Table S6:** Ampicillin kill curve of *E. coli* Δ*dcrB* with bymBDZ. bymBDZ (100 μM) was added to cultures of *E. coli* wild‐type and Δ*dcrB* in LB medium containing ampicillin (100 μg/mL). DMSO was used as a vehicle. At 0, 3, 6 and 18 h, cultures were serially diluted with 0.85% NaCl, and 10 μL aliquots were spotted in triplicate onto LB agar plates for viable cell counting. Data represents the mean ± SD of two independent experiments. Statistical analysis was performed using two‐way ANOVA in GraphPad Prism 10. *p* ≤ 0.05 (*), *p* ≤ 0.01 (**) and *p* ≤ 0.001 (***) were considered statistically significant. Fold‐change values below 0.5 were transformed and reported as −1/fold change to indicate downregulation. Data for Figure 2C and Figure S3A are provided in (A) and (B), respectively.
**Table S7:** Ampicillin kill curve of *E. coli* /pCA24N‐*dcrB. E. coli* BW25113 carrying pCA24N‐empty or pCA24N‐*dcrB* were treated with ampicillin (100 μg/mL) in LB broth. At 0, 3 and 6 h, cultures were serially diluted with 0.85% NaCl, and 10 μL aliquots were spotted in triplicate onto LB agar plates for viable cell counting. Data represents the mean ± SD of two independent experiments. Statistical analysis was performed using two‐way ANOVA in GraphPad Prism 10. *p* ≤ 0.05 (*), *p* ≤ 0.01 (**) and *p* ≤ 0.001 (***) were considered statistically significant. Fold‐change values below 0.5 were transformed and reported as −1/fold change to indicate downregulation. Data for Figure 2D and Figure S3C are provided in (A) and (B), respectively.
**Table S8:** Ampicillin kill curve of *dcrB‐*related genes. *E. coli* strains lacking or overproducing *dcrB‐*related genes were treated with ampicillin (100 μg/mL) in LB medium. At 0, 3 and 6 h, cultures were serially diluted with 0.85% NaCl, and 10 μL aliquots were spotted in triplicate onto LB agar plates for viable cell counting. Data represents the mean ± SD of two independent experiments. Statistical analysis was performed using two‐way ANOVA in GraphPad Prism 10. *p* ≤ 0.05 (*), *p* ≤ 0.01 (**) and *p* ≤ 0.001 (***) were considered statistically significant. Fold‐change values below 0.5 were transformed and reported as −1/fold change to indicate downregulation. All underlying data are consistent with the distributions shown in Figure 3B.
**Table S9:** Ampicillin kill curve of bymBDZ‐binding candidate mutants with treatment of bymBDZ. *E. coli* wild‐type or mutants were treated with ampicillin (100 μg/mL) and bymBDZ (100 μM) in LB medium. At 0, 3, 6 and 18 h, cultures were serially diluted with 0.85% NaCl, and 10 μL aliquots were spotted in triplicate onto LB agar plates for viable cell counting. Data represents the mean ± SD of two independent experiments. Statistical analysis was performed using two‐way ANOVA in GraphPad Prism 10. *p* ≤ 0.05 (*), *p* ≤ 0.01 (**) and *p* ≤ 0.001 (***) were considered statistically significant. Fold‐change values below 0.5 were transformed and reported as −1/fold change to indicate downregulation. All underlying data are consistent with the distributions shown in Figure 4B and Figure S5.
**Table S10:** Ampicillin Time‐kill curve of *E. coli* O157:H7 ATCC 43889 cells with bymBDZ. *E. coli* O157:H7 (ATCC 43889) cells were treated with ampicillin (100 μg/mL) and bymBDZ (100 μM) in LB or M9 glucose (0.4%). At 0, 3, 6 and 18 h, cultures were serially diluted with 0.85% NaCl, and 10 μL aliquots were spotted in triplicate onto LB agar plates for viable cell counting. Data represents the mean ± SD of two independent experiments. Statistical analysis was performed using two‐way ANOVA in GraphPad Prism 10. *p* ≤ 0.05 (*), *p* ≤ 0.01 (**) and *p* ≤ 0.001 (***) were considered statistically significant. Fold‐change values below 0.5 were transformed and reported as −1/fold change to indicate downregulation. All underlying data are consistent with the distributions shown in Figure 5A.
**Table S11:** Kill curves of *E. coli* BW25113 treated with bymBDZ in combination with other antibiotics. *E. coli* BW25113 cells were treated with bymBDZ (100 μM) and antibiotics (ciprofloxacin, amoxicillin, tetracycline and kanamycin) in LB or M9/glucose (0.4%). At 0, 3, 6 and 18 h, cultures were serially diluted with 0.85% NaCl, and 10 μL aliquots were spotted in triplicate onto LB agar plates for viable cell counting. Data represents the mean ± SD of two independent experiments. Statistical analysis was performed using two‐way ANOVA in GraphPad Prism 10. *p* ≤ 0.05 (*), *p* ≤ 0.01 (**) and *p* ≤ 0.001 (***) were considered statistically significant. Fold‐change values below 0.5 were transformed and reported as −1/fold change to indicate downregulation. All underlying data are consistent with the distributions shown in Figure 5B and Figure S7.
**Table S12:** MICs of WT and Δ*dcrB* in the presence of bymBDZ. *E. coli* wild‐type and Δ*dcrB* strains were grown in LB medium with varying concentrations of ampicillin in the presence of DMSO or bymBDZ. MIC_100_ was defined as the lowest concentration with complete growth inhibition and MIC_50_ was determined by linear interpolation as the concentration causing a 50% reduction in OD_600_ relative to the control (0 μg/mL ampicillin). Data represents the mean ± SD of three independent biological replicates.
**Figure S1:** Screening of compounds that promote resuscitation of *E. coli* persister cells. A. Ampicillin time‐kill curves of *E. coli* treated with another candidate compounds. *E. coli* cells were treated with ampicillin (100 μg/mL) in LB and M9/glucose (0.4%) media. DMSO was used as a vehicle. At 0, 3, 6 and 18 h, cultures were serially diluted with 0.85% NaCl, and 10 μL aliquots were spotted in triplicate onto LB agar plates for viable cell counting. Data represents the mean ± SD of two independent experiments. Statistical analysis was performed using two‐way ANOVA in GraphPad Prism 10. *p* ≤ 0.05 (*), *p* ≤ 0.01 (**) and *p* ≤ 0.001 (***) were considered statistically significant. B. Ampicillin time‐kill curves of *E. coli* treated with bymBDZ. bymBDZ (100 μM) was added to *E. coli* BW25113 culture in LB and M9/glucose (0.4%) media containing ampicillin (100 μg/mL). DMSO was used as a vehicle. At 0, 3, 6 and 18 h, cultures were serially diluted with 0.85% NaCl, and 10 μL aliquots were spotted in triplicate onto LB agar plate for viable cell counting. Data represents the mean ± SD of two independent experiments. Statistical analysis was performed using two‐way ANOVA in GraphPad Prism 10. *p* ≤ 0.05 (*), *p* ≤ 0.01 (**) and *p* ≤ 0.001 (***) were considered statistically significant. Fold‐change values below 0.5 were transformed and reported as −1/fold change to indicate downregulation.
**Figure S2:** Microscope observation of *E. coli* *MG1655 rrnbP1::GFP[ASV]‐PCA24N* persister cells at 0, 1, 3 h on M9/glucose (0.4%) agarose gel pads containing bymBDZ. Persister cells were generated as described previously and washed twice with 0.85% NaCl. The green fluorescent protein (GFP) signal of the resuscitating persisters of *E. coli* K‐12 MG1655‐ASVGFP with bymBDZ was monitored using a fluorescence microscope. *E. coli* K‐12 MG1655‐ASVGFP produces an unstable variant of GFP (half‐life < 1 h) under the control of the 16S rRNA ribosomal promoter *rrnb*P1.
**Figure S3:** Ampicillin time‐kill curves of *dcrB* mutant cells. A. Ampicillin kill curve of Δ*dcrB. E. coli* wild‐type and Δ*dcrB* cells were treated with ampicillin (100 μg/mL) in M9/glucose (0.4%) media. DMSO was used as a vehicle. At 0, 3, 6 and 18 h, cultures were serially diluted with 0.85% NaCl, and 10 μL aliquots were spotted in triplicate onto LB agar plates for viable cell counting. Data represents the mean ± SD of two independent experiments. Statistical analysis was performed using two‐way ANOVA in GraphPad Prism 10. *p* ≤ 0.05 (*), *p* ≤ 0.01 (**) and *p* ≤ 0.001 (***) were considered statistically significant. B. Ampicillin kill curve of *E. coli* /pCA24N‐*dcrB*. *E. coli* BW25113 cells carrying pCA24N‐empty or pCA24N‐*dcrB* were treated with ampicillin (100 μg/mL) in M9/glucose (0.4%) media. At 0, 3 and 6 h, cultures were serially diluted with 0.85% NaCl, and 10 μL aliquots were spotted in triplicate onto LB agar plates for viable cell counting. Data represents the mean ± SD of two independent experiments. Statistical analysis was performed using two‐way ANOVA in GraphPad Prism 10. *p* ≤ 0.05 (*), *p* ≤ 0.01 (**) and *p* ≤ 0.001 (***) were considered statistically significant. C. Ampicillin kill curve of *E. coli* /pCA24N‐*cspE*, *trpS* and *ysgA. E. coli* BW25113 cells carrying pCA24N‐empty, *cspE*, *trpS* and *ysgA* were treated with ampicillin (100 μg/mL) in M9/glucose (0.4%) media. At 0, 3 and 6 h, cultures were serially diluted with 0.85% NaCl, and 10 μL aliquots were spotted in triplicate onto LB agar plates for viable cell counting. Data represents the mean ± SD of two independent experiments. Statistical analysis was performed using two‐way ANOVA in GraphPad Prism 10. *p* ≤ 0.05 (*), *p* ≤ 0.01 (**) and *p* ≤ 0.001 (***) were considered statistically significant.
**Figure S4:** Role of *dcrB*‐related Genes in Modulating Ampicillin‐Mediated Killing in *E. coli*. *E. coli* strains with lacking or overproducing of *dcrB‐*related genes were treated with ampicillin (100 μg/mL) in M9/glucose (0.4%) media. Viable cell counts were determined at 0, 3 and 6 h.
**Figure S5:** Predicted binding model of bymBDZ to the target proteins and ampicillin time‐kill curves of its knockout strains treated with bymBDZ. A. bymBDZ binding to EmrY. B. bymBDZ binding to MdtG. C. bymBDZ binding to Tsx. D. bymBDZ binding to GlpT. Binding affinity and pose were calculated using AutoDock Vina, and the binding pocket was identified using POCOSA. The docking results were visualized using Discovery Studio. Ampicillin time–kill curves of wild‐type and knockout strains treated with bymBDZ. At 0, 3, 6 and 18 h, cultures were serially diluted with 0.85% NaCl, and 10 μL aliquots were spotted in triplicate onto LB agar plates for viable cell counting. Data represents the mean ± SD of two independent experiments. Statistical analysis was performed using two‐way ANOVA in GraphPad Prism 10. *p* ≤ 0.05 (*), *p* ≤ 0.01 (**) and *p* ≤ 0.001 (***) were considered statistically significant.
**Figure S6:** Effect of bymBDZ on Hoechst 33342 accumulation in complementary strains of Δ*mdtL*. *E. coli* Wild‐type or Δ*mdtL/*pCA24N‐empty cells turbidity at 600 nm of 0.3 were resuspended in PBS and treated with DMSO or bymBDZ (100 μM). Hoechst 33342 was added to a final concentration of 2.5 μM, and fluorescence was monitored from the top of the wells using excitation/emission wavelengths of 360/460 nm over 9 cycles at 15‐min intervals.
**Figure S7:** bymBDZ in combination antibiotics kill curves of *E. coli* BW25113 cells. *E. coli* BW25113 cells were treated with ciprofloxacin (5 μg/mL), amoxicillin (100 μg/mL), tetracycline (100 μg/mL) and bymBDZ (100 μM) in M9/glucose (0.4%) medium. At 0, 3, 6 and 18 h, cultures were serially diluted with 0.85% NaCl, and 10 μL aliquots were spotted in triplicate onto LB agar plates for viable cell counting. Data represents the mean ± SD of two independent experiments. Statistical significance was assessed using two‐way ANOVA in GraphPad Prism 10. *p* ≤ 0.05 (*), *p* ≤ 0.01 (**) and *p* ≤ 0.001 (***) were considered significant.

## Data Availability

The data supporting the findings of this study are available in the Supporting Information of this article.
