# Supplementary material for: A Novel Small Molecule Accelerates Early Persister Regrowth and Potentiates Antibiotic Killing via MdtL–DcrB
Source: Microb Biotechnol. 2026 May 3;19(5):e70368. doi: 10.1111/1751-7915.70368 (PMC13136074; doi:10.1111/1751-7915.70368)
Supplement: Supplementary file 1 — Table S1: Primers used in this study. Table S2: Ampicillin time kill curves with the candidate compounds. E. coli BW25113 cells were treated with ampicillin (100 μg/mL) and bymBDZ (100 μM) in LB or M9/glucose (0.4%). At 0, 3, 6 and 18 h, cultures were serially diluted with 0.85% NaCl, and 10 μL aliquots were spotted in triplicate onto LB agar plates for viable cell counting. Data represents the mean ± SD of two independent experiments. Statistical analysis was performed using two‐way ANOVA in GraphPad Prism 10. p ≤ 0.05 (*), p ≤ 0.01 (**) and p ≤ 0.001 (***) were considered statistically significant. Fold‐change values below 0.5 were transformed and reported as −1/fold change to indicate downregulation. Data for Figure 1B and Figure S1B are provided in (A) and data for Figure S1A is provided in (B). Table S3: Viable cell counts of pooled ASKA persister cells on M9 glucose (0.4%) agar with bymBDZ (100 μM). All ASKA clones (GFP‐) were combined, grown together to a turbidity of 2 at 600 nm LB media, and the plasmids were isolated. The pooled ASKA plasmids were electroporated into E. coli BW25113 component cells, and the cells were cultured to exponential phase. The Persister cells of pooled ASKA strains were generated by sequential rifampicin (100 μg/mL) and ampicillin (100 μg/mL) treatment, washed twice with 0.85% NaCl, and resuscitated in M9/glucose (0.4%) medium containing bymBDZ. Fold‐change value below 0.5 were transformed and reported as −1/fold change to indicate downregulation. Table S4: Resuscitation of persister cells by bymBDZ‐induced genes. Persister cells of E. coli carrying plasmids with selected genes were formed by treatment of rifampicin and ampicillin, washed twice with 0.85% NaCl, diluted to 105, and plated on M9/glucose (0.4%) agar. Viable cells of persister cells resuscitated on M9/glucose (0.4%) agar was counted. Statistical significance was determined using Student's t‐test in GraphPad Prism. p ≤ 0.05 (*), p ≤ 0.01 (**) and p ≤ 0.001 (***) were c [file MBT2-19-e70368-s001.docx]

**A novel small molecule accelerates early persister regrowth and potentiates antibiotic killing via MdtL–DcrB**

Garin Park^1^, Hyein Kim^2^, Sooyeon Song^1, 2*^

^1^ Agriculture Convergence Technology, Jeonbuk National University, 587 Baekje-Daero, Deokjin-Gu, Jeonju-Si, Jellabuk-Do, 54896, South Korea

^2^ Department of Animal Science, Jeonbuk National University, 587 Baekje-Daero, Deokjin-Gu, Jeonju-Si, Jellabuk-Do, 54896, South Korea

*For correspondence. E-mail [songsy@jbnu.ac.kr](mailto:songsy@jbnu.ac.kr)

Tel.(+)82 63-270-2606; Fax. (82) 63-270-2612

**Running title:** bymBDZ potentiates antibiotic killing and accelerates regrowth

**Keywords:** Persister cells; Early regrowth; Antibiotic potentiation; Intracellular accumulation; Envelope-associated transport; MdtL–DcrB axis

**Table S1. Primers used in this study.**

| **Gene** | **Sequence (5’-3’)** | **Product size (bp)** | **Reference** |
| --- | --- | --- | --- |
| *argT* | **F:** GTACGCCGCAGATTCTCGTT | 148 | This study |
|  | **R:** ATCCACGCCTTTACTACGCC |  |  |
| *cdd* | **F:** TTGGGCCGAAAGATCTGGAG | 127 | This study |
|  | **R:** CTGTAAGGCATGTGCGAACG |  |  |
| *cspE* | **F:** ATTACTCCGGAAGACGGCAG | 147 | This study |
|  | **R:** AGCGATTACGTTTGCAGCAG |  |  |
| *dcrB* | **F:** CGCAGCTGCAAGTGGTAAC | 183 | This study |
|  | **R:** TCTGCGCTTTTTGCTGATCG |  |  |
| *pgsA* | **F:** GGATGGCGGAGTTGGGTAAA | 157 | This study |
|  | **R:** AGTACCGCAGCCACAAAGAA |  |  |
| *yihA* | **F:** TCGCCATCCGCTGAAAGATT | 162 | This study |
|  | **R:** TCACCGTTAAACGCCAGTACA |  |  |
| *ysgA* | **F:** CACAGCTAAAAGCCGCAGTG | 138 | This study |
|  | **R:** TGCTGTTATCCTGACCACCA |  |  |
| *trpS* | **F:** CGAGAACATCAACGCTGGTC | 172 | This study |
|  | **R:** CGGCACCTTAAAGATCTCGC |  |  |
| *emrE* | **F:** ACACGGTTATGGCCATCTGT | 164 | This study |
|  | **R:** CGTTGGCCGAAAAATCCCC |  |  |
| *slp* | **F:** CCAGCCGGGGTTATATGTTGG | 136 | This study |
|  | **R:** GGCTTCAATATCAGGCTTCGC |  |  |
| *yegS* | **F:** ATTCAGGCACCACACGACAT | 126 | This study |
|  | **R:** ACGCAACAGTGGACAATCTG |  |  |
| *yhhS* | **F:** GGTTCCGCAGCAAAATCAGG | 158 | This study |
|  | **R:** ATAACGCGATTGCGACCAGT |  |  |
| *yraP* | **F:** ATTGCTATGGGCGTAGACGG | 148 | This study |
|  | **R:** TGGACGATTTCACCAGGTCG |  |  |
| *purM* | **F:** CGGTGTTGATATTGACGCGCG | 145 | (Song, et al., 2021) |
|  | **R:** CAGCACGGGTTCACGATATTTTTG |  |  |

**Table S2. Ampicillin time kill curves with the candidate compounds.** *E. coli* BW25113 cells were treated with ampicillin (100 μg/mL) and bymBDZ (100 μM) in LB or M9/glucose (0.4%). At 0, 3,6, and 18 h, cultures were serially diluted with 0.85% NaCl, and 10 μL aliquots were spotted in triplicate onto LB agar plates for viable cell counting. Data represents the mean ± SD of two independent experiments. Statistical analysis was performed using two-way ANOVA in GraphPad Prism 10. *P* ≤ 0.05 (*), *P* ≤ 0.01 (**), and *P* ≤ 0.001 (***) were considered statistically significant. Fold-change values below 0.5 were transformed and reported as −1/fold change to indicate downregulation. Data for Figure 1B and Figure S1B are provided in (A) and data for Figure S1A is provided in (B).

**(A)** **Ampicillin Time-kill curve of *E. coli* cells with bymBDZ.**

|  | 0 h | 3 h | Fold-change | 6 h | Fold-change |
| --- | --- | --- | --- | --- | --- |
| LB + amp + DMSO | 7.67E+08 ± 2.42E+08 | 4.60E+06 ± 7.82E+05 | 1 | 1.68E+04 ± 9.28E+03 | 1 |
| LB + amp +bymBDZ | 7.67E+08 ± 2.42E+08 | 2.37E+06 ± 4.23E+05*** | -1.9 | 4.00E+02 ± 8.16E+01** | -42 |
| M9/glucose (0.4%) + amp + DMSO | 7.67E+08 ± 2.42E+08 | 7.67E+04 ± 2.42E+04 | 1 | 2.00E+02 ± 1.00E+02 | 1 |
| M9/glucose (0.4%) + amp + bymBDZ | 7.67E+08 ± 2.42E+08 | 5.80E+03 ± 1.40E+03*** | -13.2 | 1.00E+02 ± 0.00E+00 | 0.5 |

**(B) Ampicillin Time-kill curve of *E. coli* cells with another compounds.** Compounds (100 μM) were added to *E. coli* cells in LB or M9/glucose (0.4%) with ampicillin (100 μg/mL). Viable cells were counted at incubation time 0, 3, 6, 18 and 24 h.

|  | LB | | | M9/glucose (0.4%) | | |
| --- | --- | --- | --- | --- | --- | --- |
| Time (h) | **+ Compound 2** | **+ Compound 3** | **+ Compound 4** | **+ Compound 2** | **+ Compound 3** | **+ Compound 4** |
| 0 | 7.67E+08 ± 2.42E+08 | | | | | |
| 3 | 3.80E+06 ± 5.83E+05 | 3.60E+06 ± 4.60E+05 | 3.15E+06 ± 6.44E+05 | 6.77E+04 ± 1.50E+04 | 3.22E+04 ± 2.07E+02 | 9.83E+04 ± 1.69E+02 |
| 6 | 2.22E+04 ± 3.43E+03 | 9.83E+03 ± 3.95E+03 | 1.12E+04 ± 4.40E+03 | 4.00E+02 ± 1.00E+02 | 2.00E+02 ± 1.00E+02 | 4.20E+02 ± 3.10E+02 |
| 18 | 6.50E+02 ± 6.61E+02 | 7.00E+02 ± 0.00E+00 | 1.00E+02 ± 0.00E+00 | 0.00E+00 ± 0.00E+00 | 0.00E+00 ± 0.00E+00 | 0.00E+00 ± 0.00E+00 |
| 24 | 0.00E+00 ± 0.00E+00 | 0.00E+00 ± 0.00E+00 | 0.00E+00 ± 0.00E+00 | 0.00E+00 ± 0.00E+00 | 0.00E+00 ± 0.00E+00 | 0.00E+00 ± 0.00E+00 |

**Table S3. Viable cell counts of pooled ASKA persister cells on M9 glucose (0.4%)** **agar with bymBDZ (100 μM).** All ASKA clones (GFP-) were combined, grown together to a turbidity of 2 at 600 nm LB media, and the plasmids were isolated. The pooled ASKA plasmids were electroporated into *E. coli* BW25113 component cells, and the cells were cultured to exponential phase. The Persister cells of pooled ASKA strains were generated by sequential rifampicin (100 μg/mL) and ampicillin (100 μg/mL) treatment, washed twice with 0.85% NaCl, and resuscitated in M9/glucose (0.4%) medium containing bymBDZ. Fold-change value below 0.5 were transformed and reported as −1/fold change to indicate downregulation.

| Media | Viable cell (CFU/mL) | Fold change |
| --- | --- | --- |
| M9/glucose (0.4%) | 1.01×10^8^ | 1 |
| M9/glucose (0.4%) + bymBDZ (100 μM) | 3.77×10^7^ | -2.7 |

**Table S4. Resuscitation of persister cells by bymBDZ-induced genes.** Persister cells of *E. coli* carrying plasmids with selected genes were formed by treatment of rifampicin and ampicillin, washed twice with 0.85% NaCl, diluted to 10^5^, and plated on M9/glucose (0.4%) agar. Viable cells of persister cells resuscitated on M9/glucose (0.4%) agar was counted. Statistical significance was determined using Student’s *t*-test in GraphPad Prism. *P* ≤ 0.05 (*), *P* ≤ 0.01 (**), and *P* ≤ 0.001 (***) were considered statistically significant. All underlying data are consistent with the distributions shown in Figure 2A.

| Plasmids | Resuscitated persisters (CFU/mL) | Fold change |
| --- | --- | --- |
| pCA24N-empty | 8.40E+07 ± 1.52E+07 | 1 |
| *argT* | 1.13E+08 ± 3.54E+06 | 1.3 |
| *cdd* | 1.34E+08 ± 9.19E+06 | 1.6 |
| *cheY* | 2.54E+08 ± 9.90E+06*** | 3.0 |
| *cspE* | 1.80E+08 ± 4.74+07* | 2.1 |
| *dcrB* | 2.79E+08 ± 7.00E+07*** | 3.3 |
| *pgsA* | 1.15E+08 ± 2.12E+07 | 1.4 |
| *trpS* | 1.71E+08 ± 1.84E+07* | 2.0 |
| *ysgA* | 1.88E+08 ±4.95E+07* | 2.2 |

**Table S5. Ribosome activation of *E. coli* by bymBDZ.** The persister cells *E. coli* MG1655 *rrnbP1*::GFP[ASV] were generated as described previously and resuscitated on M9/glucose (0.4%) agarose gel pad containing DMSO or bymBDZ (100 μM). The green fluorescent protein (GFP) signal of the resuscitating persisters was monitored. Representative images are shown in Figure S2.

| Time |  | + DMSO | + bymBDZ | *p* value  (DMSO vs bymBDZ) |
| --- | --- | --- | --- | --- |
| 0hr | Count  (Bright/ Total cells) | 148 ± 2.4 / 180.5 ± 13.9 | 149.7 ± 10.4 / 180.5 ± 4.5 | *p* = 0.89 |
|  | Bright % | 82.19% | 82.87% |  |
| 1hr | Count  (Bright/ Total cells) | 315.2 ± 230. 3 / 363.83 ± 272.7 | 195.3 ± 101. 8 / 205.5 ± 111.0 | *p* = 0.07 |
|  | Bright % | 87.48% | 95.62% |  |
| 3hr | Count  (Bright/ Total cells) | 304.8 ± 145.9 / 356.7 ± 190.0 | 248.8 ± 130.3 / 273.2 ± 149.2 | *p* = 0.37 |
|  | Bright % | 86.90% | 91.75% |  |
| 18hr | Count  (Bright/ Total cells) | 306.7 ± 67.9  / 419.8 ± 96.9 | 283.3 ± 108.4  / 454.7 ± 226.3 | *p* = 0.37 |
|  | Bright % | 73.13% | 64.35% |  |

**Table S6. Ampicillin kill curve of *E. coli*** **Δ*dcrB* with bymBDZ.** bymBDZ (100 μM) was added to cultures of *E. coli* wild-type and Δ*dcrB* in LB medium containing ampicillin (100 μg/mL). DMSO was used as a vehicle. At 0, 3, 6, and 18 h, cultures were serially diluted with 0.85% NaCl, and 10 μL aliquots were spotted in triplicate onto LB agar plates for viable cell counting. Data represents the mean ± SD of two independent experiments. Statistical analysis was performed using two-way ANOVA in GraphPad Prism 10. *P* ≤ 0.05 (*), *P* ≤ 0.01 (**), and *P* ≤ 0.001 (***) were considered statistically significant. Fold-change values below 0.5 were transformed and reported as −1/fold change to indicate downregulation. Data for Figure 2C and Figure S3A are provided in (A) and (B), respectively.

**(A) Ampicillin Time-kill curve of *E. coli* wild-type and Δ*dcrB* cells with bymBDZ in LB.**

|  | 0 h | 3 h | Fold-change | 6 h | Fold-change | 18 h | Fold-change |
| --- | --- | --- | --- | --- | --- | --- | --- |
| Wild-type | | | | | | | |
| LB + amp + DMSO | 7.33E+08 ± 1.97E+08 | 1.45E+07 ± 6.19E+06 | 1 | 3.50E+04 ± 1.05E+04 | 1 | 0.00E+00 ± 0.00E+00 | - |
| LB + amp + bymBDZ | 7.33E+08 ± 1.97E+08 | 3.98E+06 ± 1.44E+06* | -3.7 | 8.17E+03 ± 4.79E+03** | -4.3 | 0.00E+00 ± 0.00E+00 | - |
| Δ*dcrB* | | | | | | | |
| LB + amp + DMSO | 4.50E+08 ± 8.94E+06 | 2.30E+07 ± 1.04E+07 | 1 | 8.50E+04 ± 1.64E+04 | 1 | 3.83E+02 ± 1.33E+02 | 1 |
| LB + amp + bymBDZ | 4.50E+08 ± 8.94E+06 | 1.03E+07 ± 4.89E+06 | -2.27 | 6.50E+04 ± 2.07E+04 | -1.32 | 3.83E+02 ± 1.47E+02 | 1 |

**(B) Ampicillin Time-kill curve of *E. coli* wild-type and Δ*dcrB* cells with bymBDZ in M9/glucose (0.4%) media.**

|  | **0 h** | **3 h** | **Fold-change** | **6 h** | **Fold-change** | **18 h** | **Fold-change** |
| --- | --- | --- | --- | --- | --- | --- | --- |
| **Wild-type** | | | | | | | |
| **M9/glucose (0.4%) + amp + DMSO** | 7.33E+08 ± 1.97E+08 | 7.67E+04 ± 2.42E+04 | 1 | 2.50E+02 ± 7.07E+01 | 1 | 0.00E+00 ± 0.00E+00 | - |
| **M9/glucose (0.4%) + amp + bymBDZ** | 7.33E+08 ± 1.97E+08 | 5.80E+03 ± 1.64E+03** | -13.2 | 1.00E+02 ± 0.00E+00** | -2.5 | 0.00E+00 ± 0.00E+00 | - |
| **Δ*dcrB*** | | | | | | | |
| **M9/glucose (0.4%) + amp + DMSO** | 4.50E+08 ± 8.94E+06 | 5.00E+06 ± 1.31E+06 | 1 | 9.50E+02 ± 1.97E+02 | 1 | 0.00E+00 ± 0.00E+00 | - |
| **M9/glucose (0.4%) + amp + bymBDZ** | 4.50E+08 ± 8.94E+06 | 3.03E+05 ± 5.75E+04* | -16.5 | 1.60E+03 ± 4.15E+02* | 1.7 | 0.00E+00 ± 0.00E+00 | - |

**Table S7. Ampicillin kill curve of *E. coli*/pCA24N-*dcrB.*** *E. coli* BW25113 carrying pCA24N-empty or pCA24N-*dcrB* were treated with ampicillin (100 µg/mL) in LB broth. At 0, 3, and 6 h, cultures were serially diluted with 0.85% NaCl, and 10 μL aliquots were spotted in triplicate onto LB agar plates for viable cell counting. Data represents the mean ± SD of two independent experiments. Statistical analysis was performed using two-way ANOVA in GraphPad Prism 10. *P* ≤ 0.05 (*), *P* ≤ 0.01 (**), and *P* ≤ 0.001 (***) were considered statistically significant. Fold-change values below 0.5 were transformed and reported as −1/fold change to indicate downregulation. Data for Figure 2D and Figure S3C are provided in (A) and (B), respectively.

**(A) Ampicillin kill curve of *E. coli/*pCA24N-*dcrB.***

|  | **0 h** | **3 h** | **fold-change** | **6 h** | **fold-change** |
| --- | --- | --- | --- | --- | --- |
| **pCA24N/empty** | 5.74E+08 ± 1.27E+08 | 1.88E+06 ± 9.67E+05 | 1 | 1.86E+05 ± 8.66E+04 | 1 |
| **pCA24N/*dcrB*** | 5.00E+08 ± 0.00E+00 | 4.15E+05± 3.97E+05*** | -4.5 | 1.14E+04 ± 6.66E+03*** | -16.3 |

**(B) Ampicillin kill curve of *E. coli/*pCA24N-*trpS*, *cspE* and *ysgA*.**

|  | **0 h** | **3 h** | **fold-change** | **6 h** | **fold-change** |
| --- | --- | --- | --- | --- | --- |
| **pCA24N/empty** | 5.74E+08 ± 1.27E+08 | 1.88E+06 ± 9.67E+05 | 1 | 1.86E+05 ± 8.66E+04 |  |
| **pCA24N/*cspE*** | 2.55E+08 ± 5.75E+07 | 1.09E+05 ± 1.22E+05 | -44.8 | 4.17E+03 ± 9.83E+02 | -44.6 |
| **pCA24N/*trpS*** | 1.73E+08 ± 1.36E+08 | 5.65E+05 ± 4.26E+05 | 0.3 | 4.12E+05 ± 3.16E+05 | 2.2 |
| **pCA24N/*ysgA*** | 5.83E+08 ± 1.83E+08 | 4.02E+06 ± 1.71E+06 | 2.1 | 2.53E+04 ± 1.89E+04 | 0.14 |

**Table S8. Ampicillin kill curve of *dcrB-*related genes*.*** *E. coli* strains lacking or overproducing *dcrB-*related genes were treated with ampicillin (100 μg/mL) in LB medium. At 0, 3, and 6 h, cultures were serially diluted with 0.85% NaCl, and 10 μL aliquots were spotted in triplicate onto LB agar plates for viable cell counting. Data represents the mean ± SD of two independent experiments. Statistical analysis was performed using two-way ANOVA in GraphPad Prism 10. *P* ≤ 0.05 (*), *P* ≤ 0.01 (**), and *P* ≤ 0.001 (***) were considered statistically significant. Fold-change values below 0.5 were transformed and reported as −1/fold change to indicate downregulation. All underlying data are consistent with the distributions shown in Figure 3B.

**(A) Ampicillin kill curve of *dcrB*-related gene deletion mutants.**

|  | **0 h** | **3 h** | **Fold-change** | **6 h** | **Fold-change** |
| --- | --- | --- | --- | --- | --- |
| **Wild-type** | 5.85E+08 ± 2.22E+08 | 1.58E+06 ± 4.83E+05 |  | 3.80E+04 ± 1.48E+04 |  |
| **Δ*yegS*** | 5.85E+08 ± 2.22E+08 | 1.00E+04 ± 3.58E+03** | -158 | 0.00E+00 ± 0.00E+00 | ∞ |
| **Δ*yraP*** | 8.67E+08 ± 2.80E+08 | 6.75E+05 ± 3.13E+05* | -2.3 | 0.00E+00 ± 0.00E+00 | ∞ |
| **Δ*emrE*** | 9.67E+08± 3.67E+08 | 3.73E+07 ± 2.32E+07* | 23.6 | 9.75E+03 ± 2.50E+02* | -3.9 |

**(B) Ampicillin kill curve of *dcrB*-related gene overproduced mutants.**

|  | **0 h** | **3 h** | **Fold-change** | **6 h** | **Fold-change** |
| --- | --- | --- | --- | --- | --- |
| **pCA24N-empty** | 4.90E+08 ± 2.41E+08 | 1.76E+06 ± 1.13E+06 |  | 1.55E+05 ± 1.08E+05 |  |
| **pCA24N-*yegS*** | 5.00E+08 ± 1.00E+08 | 1.60E+06 ± 1.09E+06 | 0.6 | 3.33E+05 ± 1.53E+05 | 2.1 |
| **pCA24N-*yraP*** | 5.33E+08 ± 1.37E+08 | 1.72E+06 ± 1.37E+06 | 1 | 2.50E+05 ± 1.22E+05 | 1.6 |
| **pCA24N-*emrE*** | 5.83E+08 ± 1.33E+08 | 5.67E+06 ± 3.39E+06 | 3.2 | 1.27E+06 ± 1.03E+06 | 8.2 |

**Table S9.** **Ampicillin kill curve of bymBDZ-binding candidate mutants with treatment of bymBDZ.** *E. coli* wild-type or mutants were treated with ampicillin (100 μg/mL) and bymBDZ (100 μM) in LB medium. At 0, 3, 6, and 18 h, cultures were serially diluted with 0.85% NaCl, and 10 μL aliquots were spotted in triplicate onto LB agar plates for viable cell counting. Data represents the mean ± SD of two independent experiments. Statistical analysis was performed using two-way ANOVA in GraphPad Prism 10. *P* ≤ 0.05 (*), *P* ≤ 0.01 (**), and *P* ≤ 0.001 (***) were considered statistically significant. Fold-change values below 0.5 were transformed and reported as −1/fold change to indicate downregulation. All underlying data are consistent with the distributions shown in Figure 4B and Figure S5.

| **Wild-type** | | | | | | |
| --- | --- | --- | --- | --- | --- | --- |
|  | **0 h** | **3 h** | **Fold-change** | **6 h** | **Fold-change** | |
| **LB + amp**  **+ DMSO** | 8.67E+08 ± 2.07E+08 | 1.00E+07 ± 0.00E+00 | 1 | 1.97E+04 ± 1.30E+04 | 1 | |
| **LB + amp**  **+ bymBDZ** | 8.67E+08 ± 2.07E+08 | 2.94E+06 ± 3.78E+05^***^ | -3.4 | 1.80E+03 ± 1.30E+03^***^ | -10.9 | |
| **Δ*mdtL*** | | | | | |  |
|  | **0 h** | **3 h** | **Fold-change** | **6 h** | **Fold-change** | |
| **LB + amp**  **+ DMSO** | 7.80E+08 ± 2.59E+08 | 3.75E+07 ± 1.26E+07 | 1 | 3.22E+05 ± 3.65E+05 | 1 | |
| **LB + amp**  **+ bymBDZ** | 7.80E+08 ± 2.59E+08 | 1.50E+07 ± 1.00E+07 | -2.5 | 1.75E+05 ± 9.57E+04 | 0.54 | |
| **Δ*mdtG*** | | | | | |  |
|  | **0 h** | **3 h** | **Fold-change** | **6 h** | **Fold-change** | |
| **LB + amp**  **+ DMSO** | 5.75E+08 ± 3.30E+08 | 1.67E+07 ± 5.77E+06 | 1 | 1.13E+05 ± 5.77E+03 | 1 | |
| **LB + amp**  **+ bymBDZ** | 5.75E+08 ± 3.30E+08 | 2.00E+07 ± 1.41E+07 | -2.3 | 1.57E+04 ± 7.97E+03^***^ | -7.1 | |
| **Δ*emrY*** | | | | | |  |
|  | **0 h** | **3 h** | **Fold-change** | **6 h** | **Fold-change** | |
| **LB + amp**  **+ DMSO** | 7.33E+08 ± 1.75E+08 | 2.67E+07 ± 1.15E+07 | 1 | 4.03E+06 ± 3.06E+05 | 1 | |
| **LB + amp**  **+ bymBDZ** | 7.33E+08 ± 1.75E+08 | 1.75E+07 ± 5.00E+06 | 0.66 | 3.00E+05 ± 1.00E+05^*^ | -13.4 | |
| **Δ*glpT*** | | | | | |  |
|  | **0 h** | **3 h** | **Fold-change** | **6 h** | **Fold-change** | |
| **LB + amp**  **+ DMSO** | 8.33E+08 ± 4.04E+08 | 8.60E+05 ± 5.03+05 | 1 | 3.33E+02 ± 1.86E+02 | 1 | |
| **LB + amp**  **+ bymBDZ** | 8.33E+08 ± 4.04E+08 | 8.67E+05 ± 1.54E+06 | 1 | 0.00E+00 ± 0.00E+00^***^ | ∞ | |
| **Δ*tsx*** | | | | | |  |
|  | **0 h** | **3 h** | **Fold-change** | **6 h** | **Fold-change** | |
| **LB + amp**  **+ DMSO** | 6.83E+08 ± 2.93E+08 | 5.00E+06 ± 8.22+05 | 1 | 2.48E+06 ± 5.88E+05 | 1 | |
| **LB + amp**  **+ bymBDZ** | 6.83E+08 ± 2.93E+08 | 1.20E+06 ± 4.00E+05^***^ | -4.2 | 2.15E+04 ± 9.71E+03^***^ | -115.3 | |

**Table S10.** **Ampicillin Time-kill curve of *E. coli* O157:H7 ATCC 43889 cells with bymBDZ.** *E. coli* O157:H7 (ATCC 43889) cells were treated with ampicillin (100 μg/mL) and bymBDZ (100 μM) in LB or M9 glucose (0.4%). At 0, 3,6, and 18 h, cultures were serially diluted with 0.85% NaCl, and 10 μL aliquots were spotted in triplicate onto LB agar plates for viable cell counting. Data represents the mean ± SD of two independent experiments. Statistical analysis was performed using two-way ANOVA in GraphPad Prism 10. *P* ≤ 0.05 (*), *P* ≤ 0.01 (**), and *P* ≤ 0.001 (***) were considered statistically significant. Fold-change values below 0.5 were transformed and reported as −1/fold change to indicate downregulation. All underlying data are consistent with the distributions shown in Figure 5A.

|  | **0 h** | **3 h** | **Fold-change** | **6 h** | **Fold-change** | **18 h** | **Fold-change** |
| --- | --- | --- | --- | --- | --- | --- | --- |
| **LB + amp**  **+ DMSO** | 6.83E+08 ± 2.36E+07 | 1.88E+05 ± 4.71E+03 | 1 | 1.07E+04 ± 9.43E+02 | 1 | 3.17E+02 ± 7.07E+01 | 1 |
| **LB + amp**  **+ bymBDZ** | 6.83E+08 ± 2.36E+07 | 3.50E+04 ± 2.12E+04^**^ | -5.4 | 5.75E+03 ± 3.45E+02^*^ | 0.53 | 1.50E+02 ± 7.07E+01 | -2.1 |
| **M9/Glucose (0.4%) + amp + DMSO** | 6.83E+08 ± 2.36E+07 | 3.23E+05 ± 3.89E+04 | 1 | 2.88E+04 ± 1.77E+03 | 1 | 0.00E+00 ± 0.00E+00 | - |
| **M9/Glucose (0.4%) + amp + bymBDZ** | 6.83E+08 ± 2.36E+07 | 1.50E+04 ± 7.07E+03^***^ | -21.5 | 6.92E+03 ± 5.89E+02^***^ | -4.2 | 0.00E+00 ± 0.00E+00 | - |

**Table S11. Kill curves of *E. coli* BW25113 treated with bymBDZ in combination with other antibiotics.**

*E. coli* BW25113 cells were treated with bymBDZ (100 μM) and antibiotics (ciprofloxacin, amoxicillin, tetracycline, and kanamycin) in LB or M9/glucose (0.4%). At 0, 3,6, and 18 h, cultures were serially diluted with 0.85% NaCl, and 10 μL aliquots were spotted in triplicate onto LB agar plates for viable cell counting. Data represents the mean ± SD of two independent experiments. Statistical analysis was performed using two-way ANOVA in GraphPad Prism 10. *P* ≤ 0.05 (*), *P* ≤ 0.01 (**), and *P* ≤ 0.001 (***) were considered statistically significant. Fold-change values below 0.5 were transformed and reported as −1/fold change to indicate downregulation. All underlying data are consistent with the distributions shown in Figure 5B and Figure S7.

**(A) Time-kill curve of *E. coli* cells with ciprofloxacin (5 μg/mL) and bymBDZ (100 μM).**

|  | **0 h** | **3 h** | **Fold-change** | **6 h** | **Fold-change** | **18 h** | **Fold-change** |
| --- | --- | --- | --- | --- | --- | --- | --- |
| **LB + cip + DMSO** | 6.00E+08 ± 1.26E+08 | 1.73E+05 ± 5.79E+04 | 1 | 5.17E+04 ± 4.26E+04 | 1 | 0.00E+00 ± 0.00E+00 | - |
| **LB + cip + bymBDZ** | 6.00E+08 ± 1.26E+08 | 2.50E+04 ± 5.77E+03 | -6.9 | 0.00E+00 ± 0.00E+00 | -∞ | 0.00E+00 ± 0.00E+00 | - |
| **M9/Glucose (0.4%) + cip + DMSO** | 6.00E+08 ± 1.26E+08 | 6.00E+06 ± 1.08E+06 | 1 | 5.20E+06 ± 1.79E+06 | 1 | 2.67E+03 ± 1.53E+03 | 1 |
| **M9/Glucose (0.4%) + cip + bymBDZ** | 6.00E+08 ± 1.26E+08 | 2.90E+06 ± 2.97E+05 | -2.1 | 1.45E+05 ± 1.42E+05 | -35.9 | 0.00E+00 ± 0.00E+00 | -∞ |

**(B) Time-kill curve of *E. coli* cells with amoxicillin (100 μg/mL) and bymBDZ (100 μM).**

|  | **0 h** | **3 h** | **Fold-change** | **6 h** | **Fold-change** | **18 h** | **Fold-change** |
| --- | --- | --- | --- | --- | --- | --- | --- |
| **LB + amox + DMSO** | 8.00E+08 ± 1.90E+08 | 1.85E+05 ± 7.89E+04 | 1 | 1.75E+02 ± 9.57E+01 | 1 | 0.00E+00 ± 0.00E+00 | - |
| **LB + amox + bymBDZ** | 8.00E+08 ± 1.90E+08 | 5.00E+02 ± 3.16E+02 | -370 | 0.00E+00 ± 0.00E+00 | -∞ | 0.00E+00 ± 0.00E+00 | - |
| **M9/Glucose (0.4%) + amox + DMSO** | 8.00E+08 ± 1.90E+08 | 1.75E+05 ± 5.89E+04 | 1 | 2.60E+02 ± 8.94E+01 | 1 | 3.67E+02 ± 5.77E+01 | 1 |
| **M9/Glucose (0.4%) + amox + bymBDZ** | 8.00E+08 ± 1.90E+08 | 3.00E+04 ± 1.83E+04 | -5.8 | 0.00E+00 ± 0.00E+00 | -35.9 | 0.00E+00 ± 0.00E+00 | -∞ |

**(C) Time-kill curve of *E. coli* cells with tetracycline (100 μg/mL) and bymBDZ (100 μM).**

|  | **0 h** | **3 h** | **Fold-change** | **6 h** | **Fold-change** | **18 h** | **Fold-change** |
| --- | --- | --- | --- | --- | --- | --- | --- |
| **LB + tet + DMSO** | 8.00E+08 ± 1.90E+08 | 2.02E+08 ± 4.17E+07 | 1 | 7.02E+07 ± 1.57E+07 | 1 | 3.20E+07 ± 2.83E+06 | 1 |
| **LB + tet + bymBDZ** | 8.00E+08 ± 1.90E+08 | 2.05E+08 ± 3.99E+07 | 1 | 3.08E+07 ± 4.45E+06 | -2.3 | 1.60E+07 ± 3.16E+06 | -2 |
| **M9/Glucose (0.4%) + tet + DMSO** | 8.00E+08 ± 1.90E+08 | 7.00E+07 ± 1.90E+07 | 1 | 1.18E+07 ± 9.09E+06 | 1 | 3.82E+03 ± 2.26E+03 | 1 |
| **M9/Glucose (0.4%) + tet + bymBDZ** | 8.00E+08 ± 1.90E+08 | 2.60E+07 ± 1.14E+07 | -2.7 | 9.92E+05 ± 5.14E+05 | -11.9 | 4.78E+03 ± 1.95E+03 | 1 |

**(D) Time-kill curve of *E. coli* cells with kanamycin (200 μg/mL) and bymBDZ (100 μM).**

|  | **0 h** | **3 h** | **Fold-change** | **6 h** | **Fold-change** | **18 h** | **Fold-change** |
| --- | --- | --- | --- | --- | --- | --- | --- |
| **LB + kan + DMSO** | 6.00E+08 ± 1.26E+08 | 1.53E+06 ± 3.51E+05 | 1 | 0.00E+00 ± 0.00E+00 | - | 0.00E+00 ± 0.00E+00 | - |
| **LB + kan + bymBDZ** | 6.00E+08 ± 1.26E+08 | 1.28E+06 ± 3.59E+05 | 0.8 | 0.00E+00 ± 0.00E+00 | - | 0.00E+00 ± 0.00E+00 | - |
| **M9/Glucose (0.4%) + kan + DMSO** | 6.00E+08 ± 1.26E+08 | 0.00E+00 ± 0.00E+00 | - | 0.00E+00 ± 0.00E+00 | - | 0.00E+00 ± 0.00E+00 | - |
| **M9/Glucose (0.4%) + kan + bymBDZ** | 6.00E+08 ± 1.26E+08 | 0.00E+00 ± 0.00E+00 | - | 0.00E+00 ± 0.00E+00 | - | 0.00E+00 ± 0.00E+00 | - |

**Table S12. MICs of WT and Δ*dcrB* in the presence of bymBDZ.** *E. coli* wild-type and Δ*dcrB* strains were grown in LB medium with varying concentrations of ampicillin in the presence of DMSO or bymBDZ. MIC_100_ was defined as the lowest concentration with complete growth inhibition and MIC_50_ was determined by linear interpolation as the concentration causing a 50% reduction in OD₆₀₀ relative to the control (0 μg/mL ampicillin). Data represents the mean ± SD of three independent biological replicates.

| **Treatment** | **DMSO** | | **bymBDZ** | |
| --- | --- | --- | --- | --- |
| **Strain** | **MIC_100_**  **(μg/mL)** | **MIC_50_**  **Avg (μg/mL)** | **MIC_100_**  **(μg/mL)** | **MIC_50_**  **Avg (μg/mL)** |
| **Wild-type** | 8 | 3.72 ± 1.15 | 4 | 2.37±0.30 |
| **Δ*dcrB*** | 8 | 3.35 ± 0.81 | 4 | 2.34 ± 0.32 |

**Supplementary figures**

**Figure S1. Screening of compounds that promote resuscitation of *E. coli* persister cells.**

**A.** **Ampicillin time-kill curves of *E. coli* treated with another candidate compounds.** *E. coli* cells were treated with ampicillin (100 µg/mL) in LB and M9/glucose (0.4%) media. DMSO was used as a vehicle. At 0, 3, 6, and 18 h, cultures were serially diluted with 0.85% NaCl, and 10 μL aliquots were spotted in triplicate onto LB agar plates for viable cell counting. Data represents the mean ± SD of two independent experiments. Statistical analysis was performed using two-way ANOVA in GraphPad Prism 10. P ≤ 0.05 (*), P ≤ 0.01 (**), and *P* ≤ 0.001 (***) were considered statistically significant.

**B. Ampicillin time-kill curves of *E. coli* treated with bymBDZ.** bymBDZ (100 μM) was added to *E. coli* BW25113 culture in LB and M9/glucose (0.4%) media containing ampicillin (100 μg/mL). DMSO was used as a vehicle. At 0, 3, 6, and 18 h, cultures were serially diluted with 0.85% NaCl, and 10 μL aliquots were spotted in triplicate onto LB agar plate for viable cell counting. Data represents the mean ± SD of two independent experiments. Statistical analysis was performed using two-way ANOVA in GraphPad Prism 10. *P* ≤ 0.05 (*), *P* ≤ 0.01 (**), and *P* ≤ 0.001 (***) were considered statistically significant. Fold-change values below 0.5 were transformed and reported as −1/fold change to indicate downregulation.

**Figure S2. Microscope observation of *E. coli MG1655 rrnbP1::GFP[ASV]-PCA24N* persister cells at 0, 1, 3 h on M9/glucose (0.4%) agarose gel pads containing bymBDZ.** Persister cells were generated as described previously and washed twice with 0.85% NaCl. The green fluorescent protein (GFP) signal of the resuscitating persisters of *E. coli* K-12 MG1655-ASVGFP with bymBDZ was monitored using a fluorescence microscope. *E. coli* K-12 MG1655-ASVGFP produces an unstable variant of GFP (half-life less than 1 hr) under the control of the 16S rRNA ribosomal promoter *rrnb*P1.

**Figure S3. Ampicillin time-kill curves of *dcrB* mutant cells.**

**A. Ampicillin kill curve of Δ*dcrB.*** *E. coli* wild-type and Δ*dcrB* cells were treated with ampicillin (100 µg/mL) in M9/glucose (0.4%) media. DMSO was used as a vehicle. At 0, 3, 6, and 18 h, cultures were serially diluted with 0.85% NaCl, and 10 μL aliquots were spotted in triplicate onto LB agar plates for viable cell counting. Data represents the mean ± SD of two independent experiments. Statistical analysis was performed using two-way ANOVA in GraphPad Prism 10. *P* ≤ 0.05 (*), *P* ≤ 0.01 (**), and *P* ≤ 0.001 (***) were considered statistically significant.

**B. Ampicillin kill curve of *E. coli*/pCA24N-*dcrB*.** *E. coli* BW25113 cells carrying pCA24N-empty or pCA24N-*dcrB* were treated with ampicillin (100 µg/mL) in M9/glucose (0.4%) media. At 0, 3, and 6 h, cultures were serially diluted with 0.85% NaCl, and 10 μL aliquots were spotted in triplicate onto LB agar plates for viable cell counting. Data represents the mean ± SD of two independent experiments. Statistical analysis was performed using two-way ANOVA in GraphPad Prism 10. *P* ≤ 0.05 (*), *P* ≤ 0.01 (**), and *P* ≤ 0.001 (***) were considered statistically significant.

**C. Ampicillin kill curve of *E. coli*/pCA24N-*cspE, trpS,* and *ysgA.*** *E. coli* BW25113 cells carrying pCA24N-empty, *cspE, trpS,* and *ysgA* were treated with ampicillin (100 µg/mL) in M9/glucose (0.4%) media. At 0, 3, and 6 h, cultures were serially diluted with 0.85% NaCl, and 10 μL aliquots were spotted in triplicate onto LB agar plates for viable cell counting. Data represents the mean ± SD of two independent experiments. Statistical analysis was performed using two-way ANOVA in GraphPad Prism 10. *P* ≤ 0.05 (*), *P* ≤ 0.01 (**), and *P* ≤ 0.001 (***) were considered statistically significant.

**Figure S4. Role of *dcrB*-related Genes in Modulating Ampicillin-Mediated Killing in *E. coli.*** *E. coli* strains with lacking or overproducing of *dcrB-*related genes were treated with ampicillin (100 μg/mL) in M9/glucose (0.4%) media. Viable cell counts were determined at 0, 3, and 6 h.

**Figure S5. Predicted binding model of bymBDZ to the target proteins and ampicillin time-kill curves of its knockout strains treated with bymBDZ.**

**A.** **bymBDZ binding to EmrY.**

**B. bymBDZ binding to MdtG.**

**C. bymBDZ binding to Tsx.**

**D. bymBDZ binding to GlpT.**

Binding affinity and pose were calculated using AutoDock Vina, and the binding pocket was identified using POCOSA. The docking results were visualized using Discovery Studio. Ampicillin time–kill curves of wild-type and knockout strains treated with bymBDZ. At 0, 3, 6, and 18 h, cultures were serially diluted with 0.85% NaCl, and 10 μL aliquots were spotted in triplicate onto LB agar plates for viable cell counting. Data represents the mean ± SD of two independent experiments. Statistical analysis was performed using two-way ANOVA in GraphPad Prism 10. *P* ≤ 0.05 (*), *P* ≤ 0.01 (**), and *P* ≤ 0.001 (***) were considered statistically significant.

**Figure S6. Effect of bymBDZ on Hoechst 33342 accumulation in complementary strains of Δ*mdtL*.** *E. coli* Wild-type or Δ*mdtL/*pCA24N-empty cells turbidity at 600 nm of 0.3 were resuspended in PBS and treated with DMSO or bymBDZ (100 μM). Hoechst 33342 was added to a final concentration of 2.5 μM, and fluorescence was monitored from the top of the wells using excitation/emission wavelengths of 360/460 nm over 9 cycles at 15-min intervals.

**Figure S7. bymBDZ in combination antibiotics kill curves of *E. coli* BW25113 cells.** *E. coli* BW25113 cells were treated with ciprofloxacin (5 µg/mL), amoxicillin (100 µg/mL), tetracycline (100 µg/mL) and bymBDZ (100 µM) in M9/glucose (0.4%) medium. At 0, 3,6, and 18 h, cultures were serially diluted with 0.85% NaCl, and 10 μL aliquots were spotted in triplicate onto LB agar plates for viable cell counting. Data represents the mean ± SD of two independent experiments. Statistical significance was assessed using two-way ANOVA in GraphPad Prism 10. *P* ≤ 0.05 (*), *P* ≤ 0.01 (**), and *P* ≤ 0.001 (***) were considered significant.

**
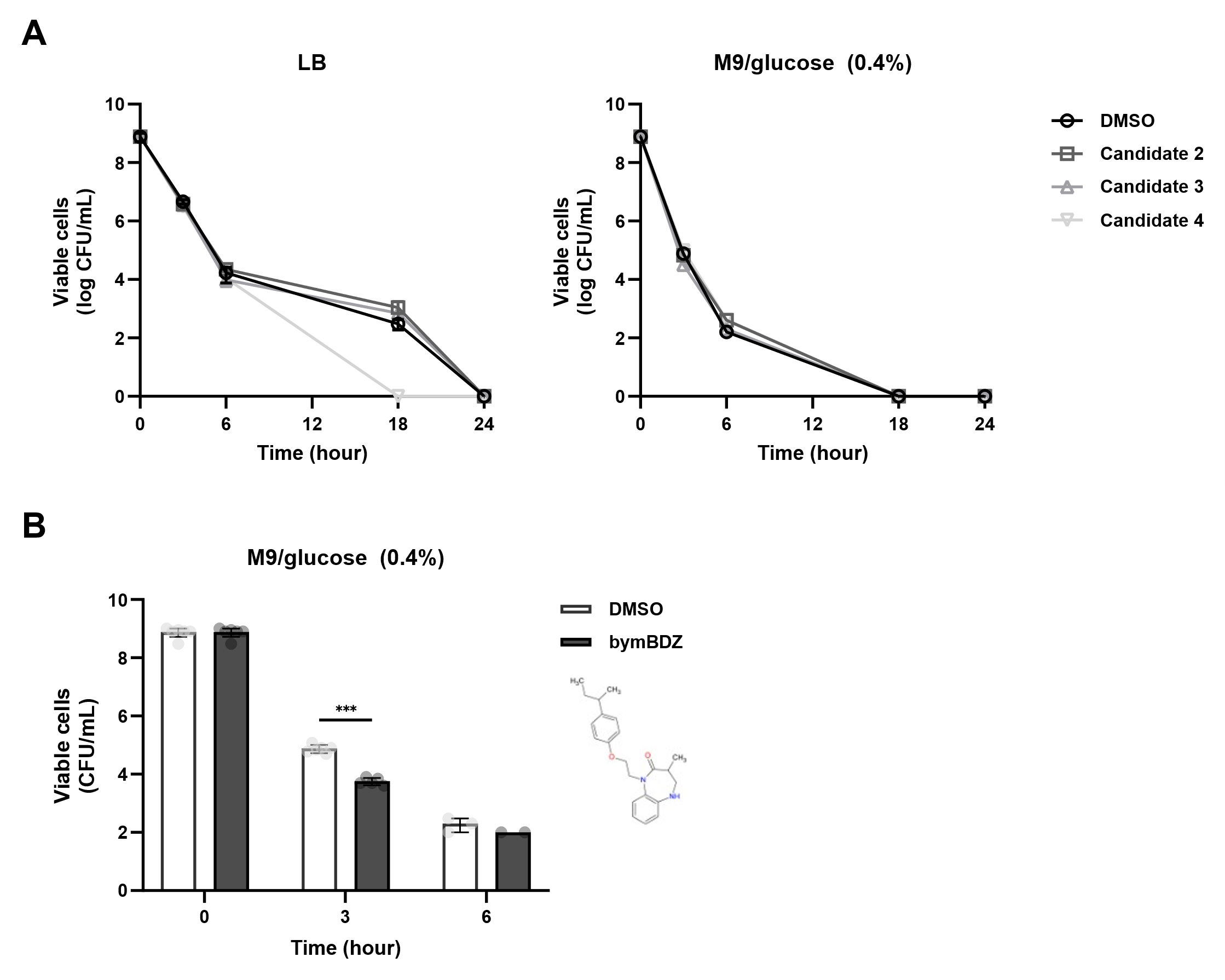
**

**Figure S1**

**
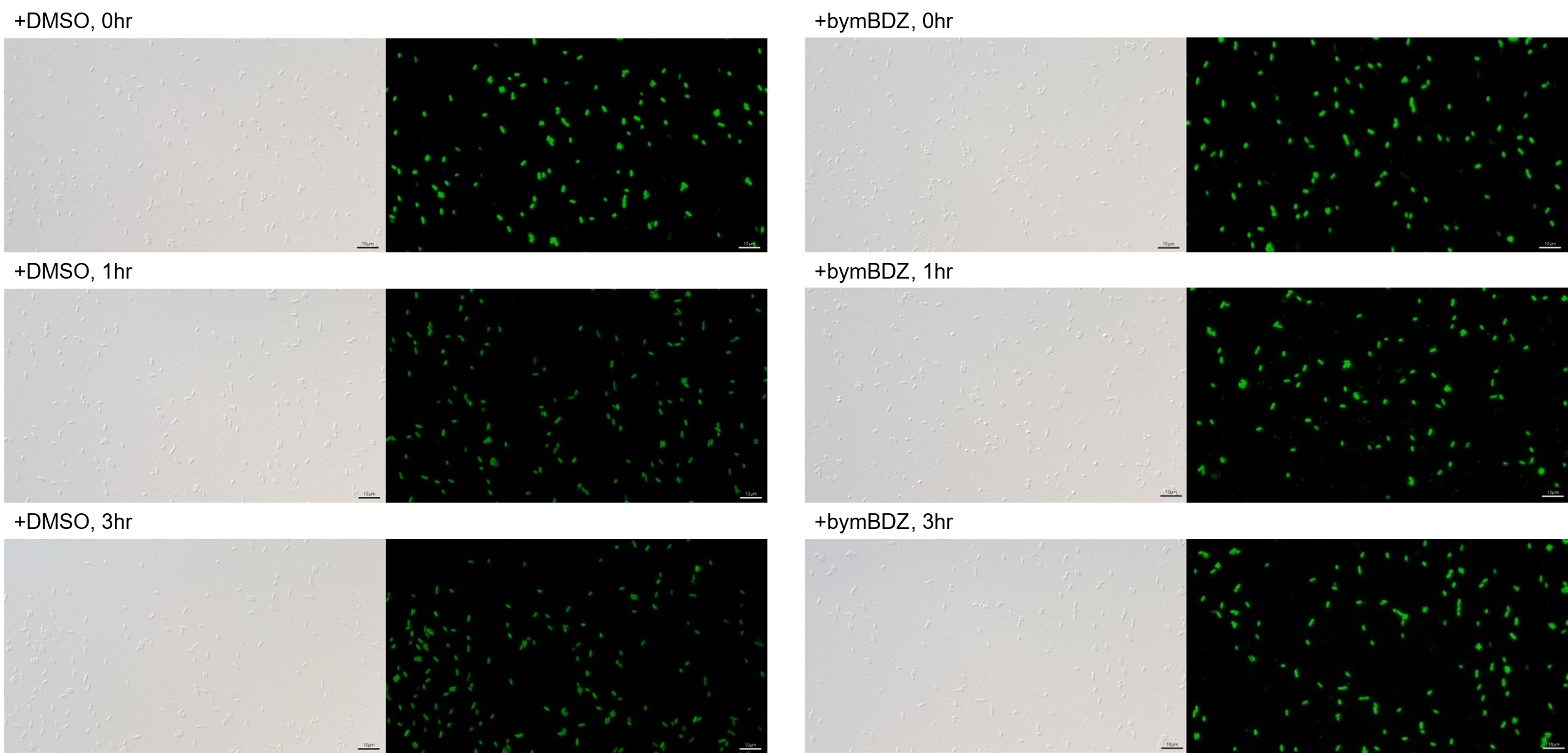
**

**Figure S2**

**
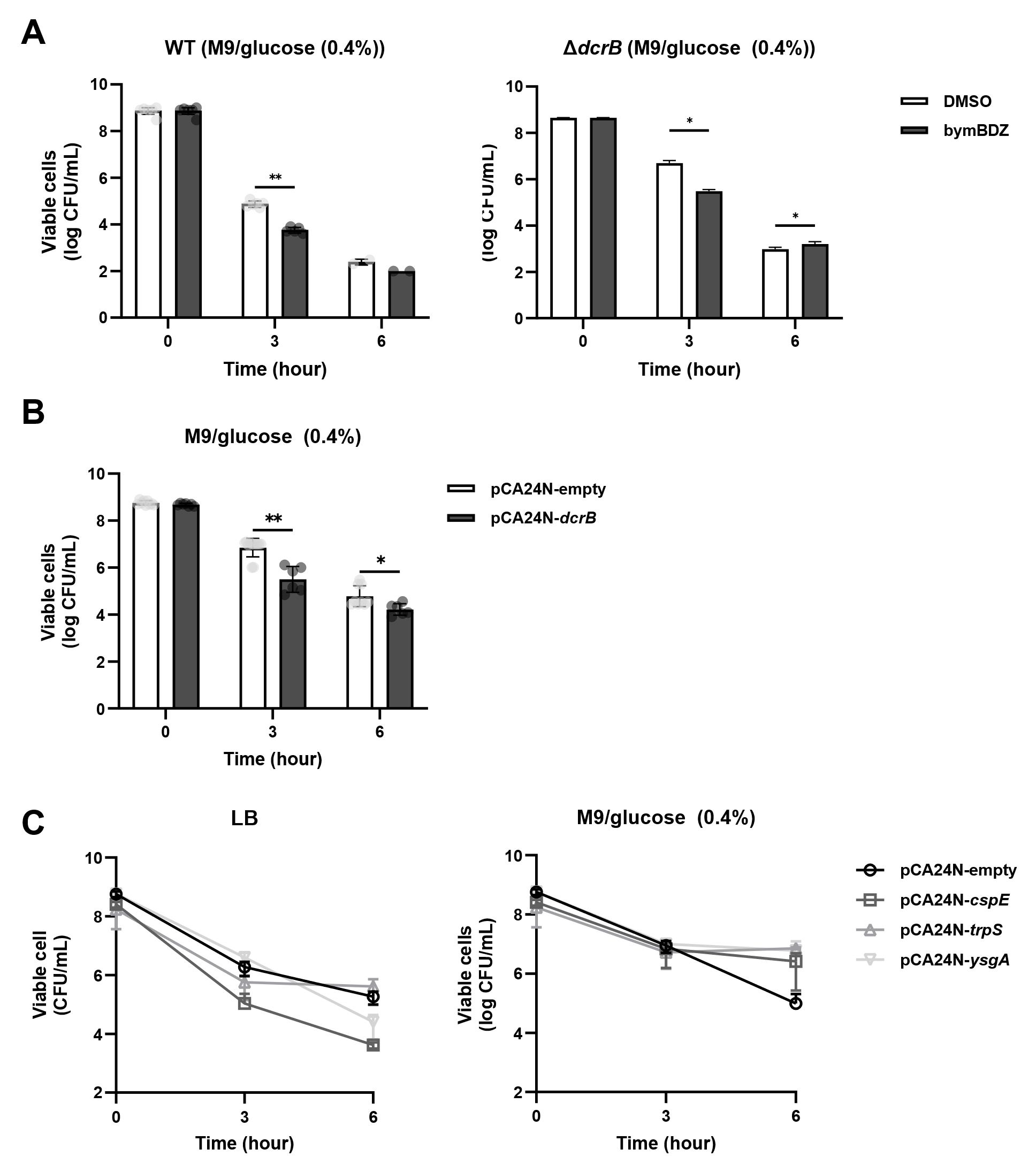
**

**Figure S3**

**
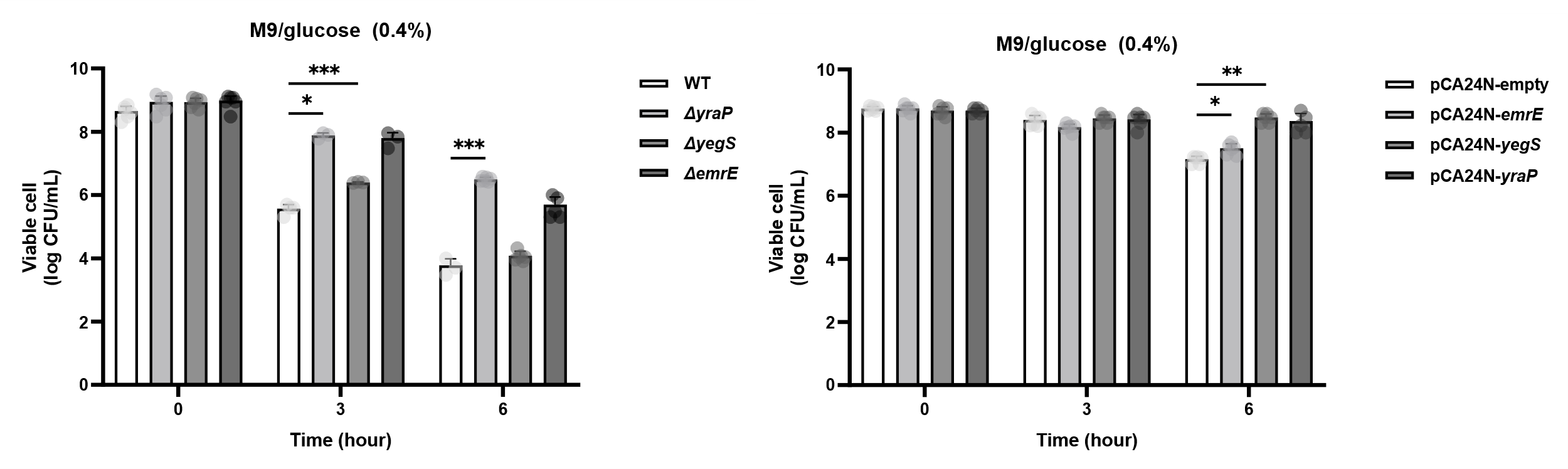
**

**Figure S4**

**
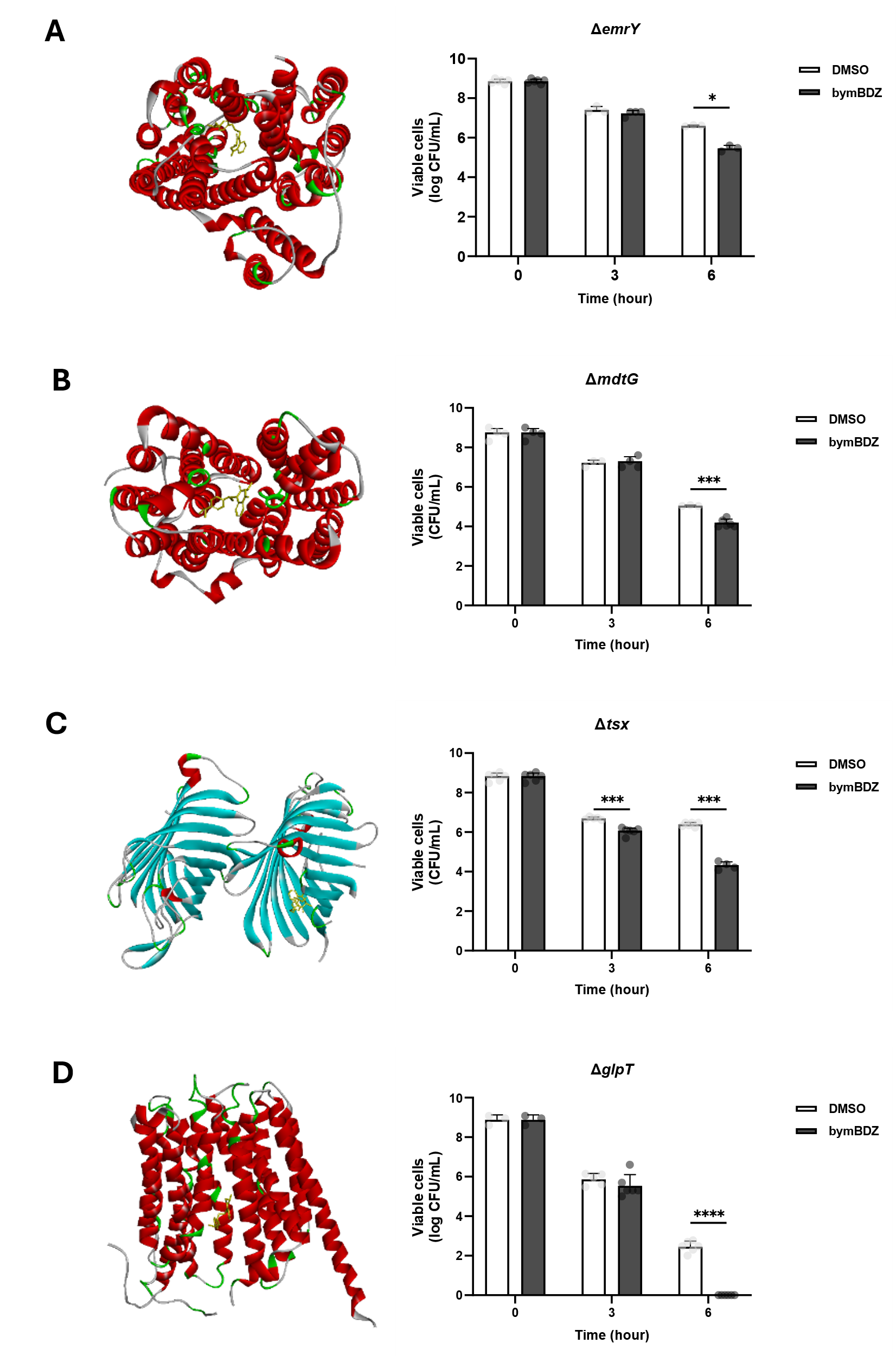
**

**Figure S5**

**
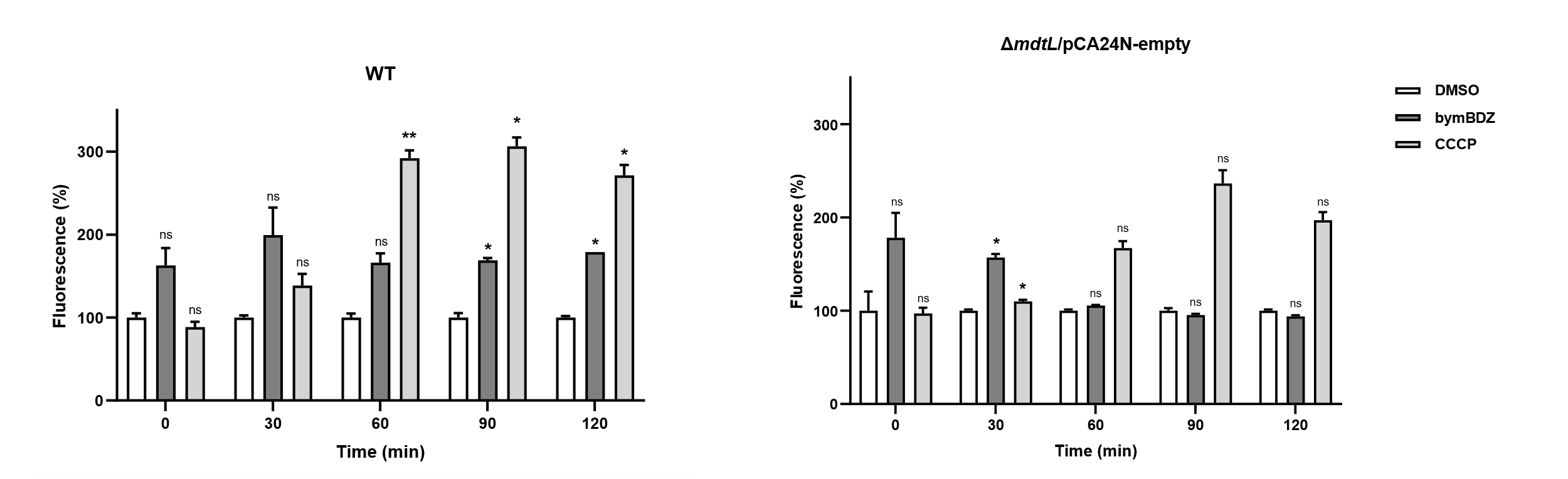
**

**Figure S6**

**
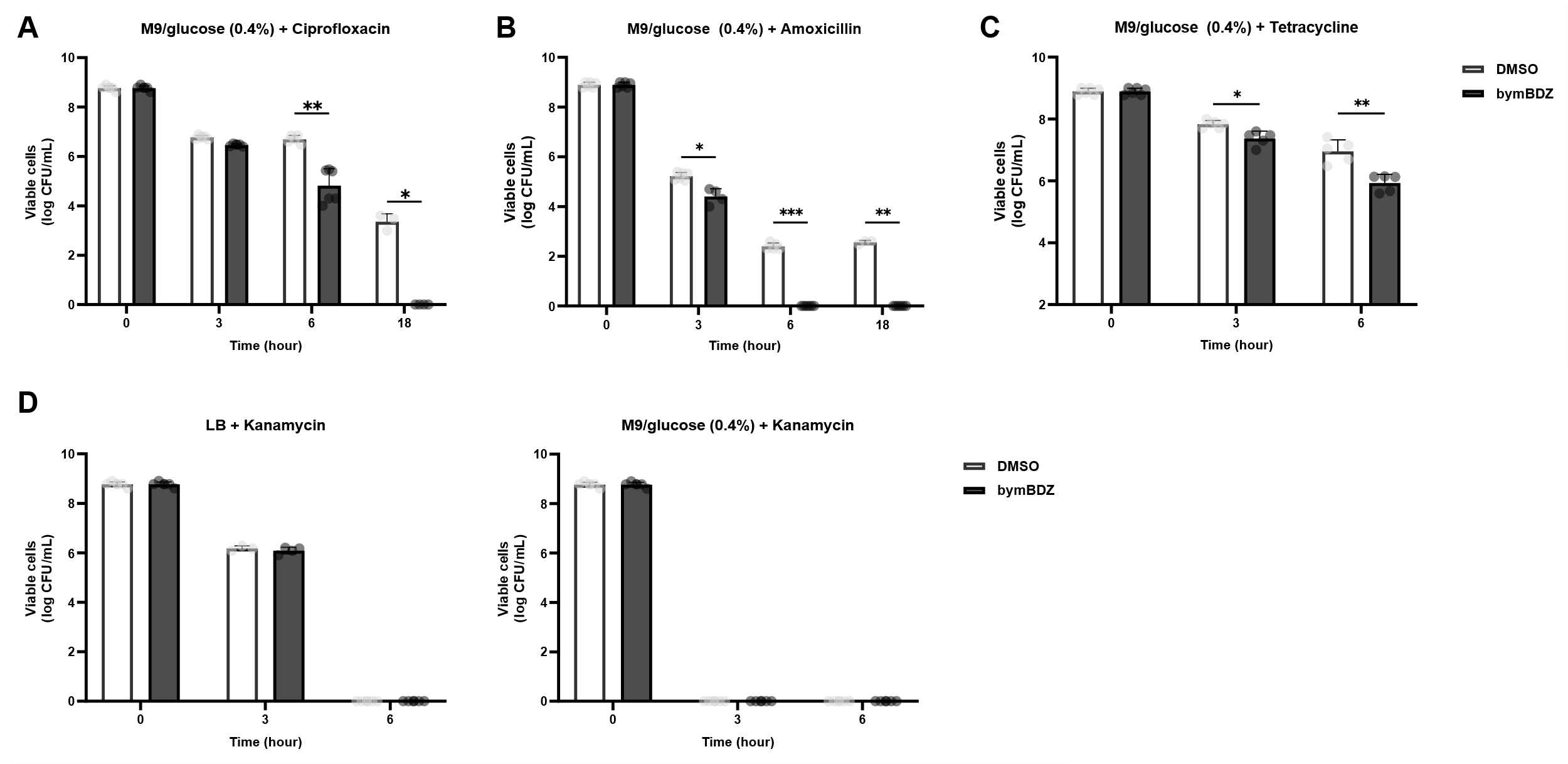
**

**Figure S7**

**References**

Song, S., Kim, J.S., Yamasaki, R., Oh, S., Benedik, M.J., and Wood, T.K. (2021) *Escherichia coli* cryptic prophages sense nutrients to influence persister cell resuscitation, *Environmental Microbiology* **23**: 7245–7254.
